# Pterosin B prevents chondrocyte hypertrophy and osteoarthritis in mice by inhibiting Sik3

**DOI:** 10.1038/ncomms10959

**Published:** 2016-03-24

**Authors:** Yasuhito Yahara, Hiroshi Takemori, Minoru Okada, Azuma Kosai, Akihiro Yamashita, Tomohito Kobayashi, Kaori Fujita, Yumi Itoh, Masahiro Nakamura, Hiroyuki Fuchino, Nobuo Kawahara, Naoshi Fukui, Akira Watanabe, Tomoatsu Kimura, Noriyuki Tsumaki

**Affiliations:** 1 Department of Cell Growth and Differentiation, Center for iPS Cell Research and Application, Kyoto University, 53 Kawahara-cho, Shogoin, Sakyo-ku, Kyoto 606-8507, Japan; 2 Department of Orthopaedic Surgery, Faculty of Medicine, University of Toyama, 2630, Sugitani, Toyama 930-0194, Japan; 3 Laboratory of Cell Signaling and Metabolic Disease, National Institutes of Biomedical Innovation, Health and Nutrition, 7-6-8, Asagi, Saito, Ibaraki, Osaka 567-0085, Japan; 4 Genome/Epigenome Analysis Core Facility, Center for iPS Cell Research and Application, Kyoto University, 53 Kawahara-cho, Shogoin, Sakyo-ku, Kyoto 606-8507, Japan; 5 Research Center for Medicinal Plant Resources, Tsukuba Division, National Institutes of Biomedical Innovation, Health and Nutrition, 1-2, Hachimandai, Tsukuba, Ibaraki 305-0843, Japan; 6 Graduate School of Arts and Sciences, Department of Life Sciences, The University of Tokyo, Komaba 3-8-1, Meguro-ku, Tokyo 153-8902, Japan

## Abstract

Osteoarthritis is a common debilitating joint disorder. Risk factors for osteoarthritis include age, which is associated with thinning of articular cartilage. Here we generate chondrocyte-specific salt-inducible kinase 3 (Sik3) conditional knockout mice that are resistant to osteoarthritis with thickened articular cartilage owing to a larger chondrocyte population. We also identify an edible *Pteridium aquilinum* compound, pterosin B, as a Sik3 pathway inhibitor. We show that either Sik3 deletion or intraarticular injection of mice with pterosin B inhibits chondrocyte hypertrophy and protects cartilage from osteoarthritis. Collectively, our results suggest Sik3 regulates the homeostasis of articular cartilage and is a target for the treatment of osteoarthritis, with pterosin B as a candidate therapeutic.

## Abstract (web summary)

Therapies are needed for the prevention of chondrocyte hypertrophy and thinning of articular cartilage, features of osteoarthritic joint destruction. Here, the authors show that interfering with Sik3 signalling can increase the size of the chondrocyte population and reduce severity of a surgically induced mouse model of osteoarthritis.

## Graphical abstract

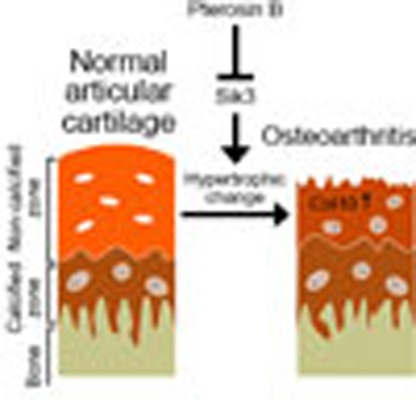

Articular cartilage lubricates the ends of bones. Its loss can ultimately result in osteoarthritis, a common debilitating joint disorder that affects more than 15% of the population over the age of 60 (refs 1, 2). Common risk factors for osteoarthritis include age, excess mechanical loading and metabolic syndrome. The most apparent age-associated change is the reduced thickness of articular cartilage^3^,^4^; however, whether thinner cartilage is more susceptible to osteoarthritis is unclear. Although extensive studies have revealed age-related molecular changes in chondrocytes, the molecules that control thickness of articular cartilage are unknown.

During development, epiphyseal cartilage at both ends of the bone gives rise to articular cartilage and growth plate cartilage after the formation of the secondary ossification centre. Chondrocytes express type II collagen (COL2) and type XI collagen (COL11) during proliferation, but will ultimately stop proliferation and undergo hypertrophy in epiphyseal cartilage. Hypertrophic chondrocytes express type X collagen (COL10) and matrix metalloproteinases (MMPs), which contribute to the degradation of cartilage and replacement by bone. Articular cartilage consists of two layers, a non-calcified zone and a calcified zone, which are separated by the tidemark. Chondrocytes in the non-calcified zone express COL2 and COL11, whereas chondrocytes in the calcified zone express COL10. However, chondrocytes in the non-calcified zone express COL10 and MMPs in osteoarthritic conditions^5^,^6^,^7^, suggesting activation of the hypertrophy programme. Inhibiting these hypertrophic changes has been shown to reduce the severity of cartilage lesions in mouse osteoarthritis models^6^,^8^,^9^. Thus, molecules that activate the hypertrophy programme might be promising therapeutic targets for osteoarthritis drugs.

We previously reported that Sik3 knockout mice have delayed chondrocyte hypertrophy in mice^10^. Here we show that SIK3 is activated in human osteoarthritic cartilage, leading us to hypothesize that Sik3 is important for the development of osteoarthritis. However, Sik3 total knockout mice have severely malformed skeletons and most die at birth^10^; therefore, we generated tamoxifen conditional chondrocyte-specific Sik3 knockout mice. Deletion of Sik3 in adult mice causes thickening of articular cartilage and resistance to surgically induced osteoarthritis, a phenotype associated with reduced expression of Col10 in the non-calcified zone of articular cartilage. We screen for compounds that inhibit SIK3 activity, identifying pterosin B as a suppressor of Sik3 signalling. Intraarticular administration of pterosin B protects the mice from osteoarthritic change and Col10 expression. These results suggest that pterosin B is a promising candidate for osteoarthritis drug development.

## Results

### Expression of phospho-SIK3 in osteoarthritic cartilage

We investigated the expression of total SIK3 and phosphorylated SIK3 at T163 (pSIK3), which is the active form of SIK3 (refs 11, 12), in articular cartilage. Immunohistochemical analysis revealed that total SIK3 was uniformly detected from the surface to deep layer, whereas pSik3 was detected only in the deep layer of normal articular cartilage (Supplementary Fig. 1a). On the other hand, pSIK3 was expressed in the superficial layer of severely affected osteoarthritic articular cartilage (Supplementary Fig. 1b). Moreover, the worse the OARSI grade^13^, the more cells in the superficial layers expressed pSIK3. These results suggest that activation of SIK3 is related to osteoarthritis development.

### Generation of inducible Sik3 conditional knockout mice

We generated transgenic mice bearing the *Col11a2-CreER* construct, in which the *CreER* gene is linked to chondrocyte-specific α2(XI) collagen chain gene (*Col11a2*) promoter/enhancer sequences (Supplementary Fig. 2a). We mated *Col11a2-CreER* transgenic mice with *Rosa26-stop*^*flox*^*-EYFP* tester mice to prepare *Col11a2-CreER*; *Rosa26-stop*^*flox*^*-EYFP* mice. On intraperitoneal injection of tamoxifen, *Col11a2-CreER*; *Rosa26-stop*^*flox*^*-EYFP* mice showed YFP fluorescence exclusively in primordial cartilage in the limbs and ribs of embryos at 14.5 days postcoitum, whereas *Rosa26-stop*^*flox*^*-EYFP* mice showed no YFP fluorescence (Supplementary Fig. 2b). *Col11a2-CreER*; *Rosa26-stop*^*flox*^*-EYFP* mice treated with tamoxifen showed YFP expression exclusively in articular cartilage in knee joints at 16 weeks of age, whereas mice without tamoxifen treatment showed no YFP expression (Supplementary Fig. 2c). These results suggest that the *Col11a2-CreER* transgenic allele can induce recombination at *loxP* sites specifically in chondrocytes in a tamoxifen-dependent manner.

We next generated *Sik3*^*flox/+*^ (Supplementary Fig. 3a) mice and prepared *Col11a2-CreER; Sik3*^*flox/flox*^ mice. *Col11a2-CreER; Sik3*^*flox/+*^ mice and *Sik3*^*flox/flox*^ mice were used as controls. We confirmed that Sik3 was deleted in the chondrocytes of *Col11a2-CreER; Sik3*^*flox/flox*^ mice after administration of tamoxifen (Supplementary Fig. 3b). Hereafter, we call tamoxifen-treated *Col11a2-CreER; Sik3*^*flox/flox*^ mice as *Sik3*^*Δ/Δ*^ mice, tamoxifen-treated *Col11a2-CreER; Sik3*^*flox/+*^ mice as *Sik3*^*Δ/+*^ mice and tamoxifen-treated *Sik3*^*flox/flox*^ mice as *Sik3*^*f/f*^ mice.

### Deletion of Sik3 increases thickness of articular cartilage

**Figure 1:**
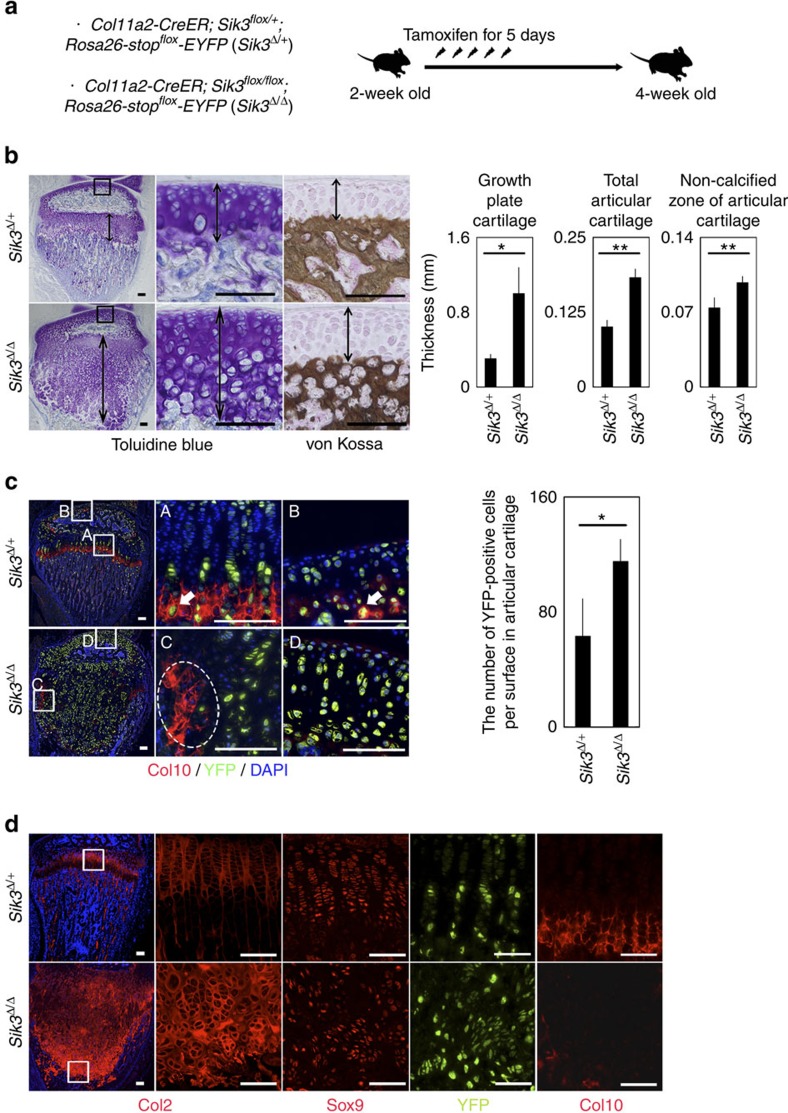
Phenotype of cartilage in mice lacking Sik3 after birth. (**a**) We injected *Col11a2-CreER; Sik3*^*flox/flox*^; *Rosa26-stop*^*flox*^*-EYFP* mice and *Col11a2-CreER; Sik3*^*flox/+*^; *Rosa26-stop*^*flox*^*-EYFP* mice with tamoxifen daily for 5 consecutive days beginning at 2 weeks of age and killed them at 4 weeks of age. (**b**) Semiserial histological sagittal sections of proximal tibia stained with toluidine blue and von Kossa. Boxed regions are shown to the right. Thickness of growth plate cartilage, total articular cartilage and non-calcified articular cartilage at the vertex of the articular surface in the corresponding sections of the weight-bearing area (indicated by arrows) were measured and are shown in the right graphs. *n*=4 mice. (**c**) Semiserial sections of **b** were immunostained for Col10 (red) and YFP (yellow). Blue colour is DAPI. Boxed regions indicated by A–D are shown to the right. The numbers of EYFP-positive cells per 360 μm-surface in articular cartilage were counted and are shown in the right graph. *Arrows*, *Col11a2*-marked YFP-positive chondrocytes in Col10-positive matrix in *Sik3*^*Δ/+*^ mice. *Dotted circle*, the Col10-positive area lacking YFP-positive cells in *Sik3*^*Δ/Δ*^ mice; *n*=4 mice. (**d**) Semiserial sections of **b**,**c** were immunostained for Col2, Sox9, YFP and Col10. Boxed regions in the left panels (Col2) are shown in the right panels. The images are representative of two independent experiments. Error bars denote means±s.d. *P<0.05 and **P<0.01 by the *t*-test. Scale bars, 100 μm.

To analyse Sik3 function in articular cartilage after birth, we treated mice with tamoxifen at 2 weeks of age and killed them at 4 weeks of age (Fig. 1a). To trace the fate of *Sik3*^*Δ/Δ*^ cells, we performed this experiment on the *Rosa26-stop*^*flox*^*-EYFP* background (*Col11a2-CreER; Sik3*^*flox/flox*^; *Rosa26-stop*^*flox*^*-EYFP* mice). Histological sections were stained with toluidine blue and von Kossa to identify total articular cartilage (non-calcified zone and calcified zone) and the non-calcified zone only. The thicknesses of total articular cartilage and the non-calcified zone of articular cartilage as well as that of growth plate cartilage were all increased in *Sik3*^*Δ/Δ*^ mice compared with *Sik3*^*Δ/+*^ mice (Fig. 1b). In addition, the numbers of YFP-positive cells per surface in articular cartilage and growth plate cartilage in *Sik3*^*Δ/Δ*^ mice were increased compared with those in *Sik3*^*Δ/+*^ mice (Fig. 1c). The expression levels of *Col2a1* and *Acan* did not differ between pellets of *Col11a2-CreER; Sik3*^*flox/flox*^ chondrocytes cultured in the presence of tamoxifen and those in the absence of tamoxifen (Supplementary Fig. 3c), suggesting that the amount of matrix production per chondrocyte did not change. These results collectively suggest that Sik3 deletion increased the population of chondrocyte progenies, resulting in increased total amount of matrix and increased cartilage thickness.

We hypothesized that the increased chondrocyte population in *Sik3*^*Δ/Δ*^ mice might be caused either by increased proliferation rates or decreased removal rates of the cells. To analyse the proliferation rates, we performed BrdU labelling and found that Sik3 deletion had no effect on either articular or growth plate cartilage (Supplementary Fig. 4a). BrdU-positive cells were absent in articular cartilage in both *Sik3*^*Δ/Δ*^ mice and *Sik3*^*Δ/+*^ mice for the entire labelling period (3 h). On the other hand, immunohistochemical analysis showed that deletion of Sik3 almost eliminated Col10 expression in articular and growth plate cartilage (Fig. 1c), suggesting that the removal of chondrocytes was compromised. Lineage tracing revealed that progenies of *Col11a2*-marked YFP-positive chondrocytes expressed Col10 in the hypertrophic zone of growth plate cartilage (Fig. 1cA, arrow) and in the calcified zone of articular cartilage (Fig. 1cB, arrow) in control mice, but that Sik3-deficient chondrocytes did not express Col10 in either (Fig. 1cC and 1cD). Moreover, YFP-positive cells were absent from the Col10-positive area in *Sik3*^*Δ/Δ*^ mice (Fig. 1cC, dotted circle), suggesting that progenies of *Sik3*^*Δ/Δ*^ chondrocytes continued to express Col2 instead. We confirmed that Sik3-deficient cells expressed Col2 and Sox9, but not Col10 in growth plate cartilage (Fig. 1d, bottom panels), indicating that Sik3-deficient cells remain chondrocytes. These results collectively suggest that Sik3 deletion keeps the Col2-positive; Col10-negative chondrocyte state, which increases the population of Col2-positive; Col10-negative chondrocytes and thickening of articular cartilage.

### Sik3 deletion protects cartilage from osteoarthritis

**Figure 2:**
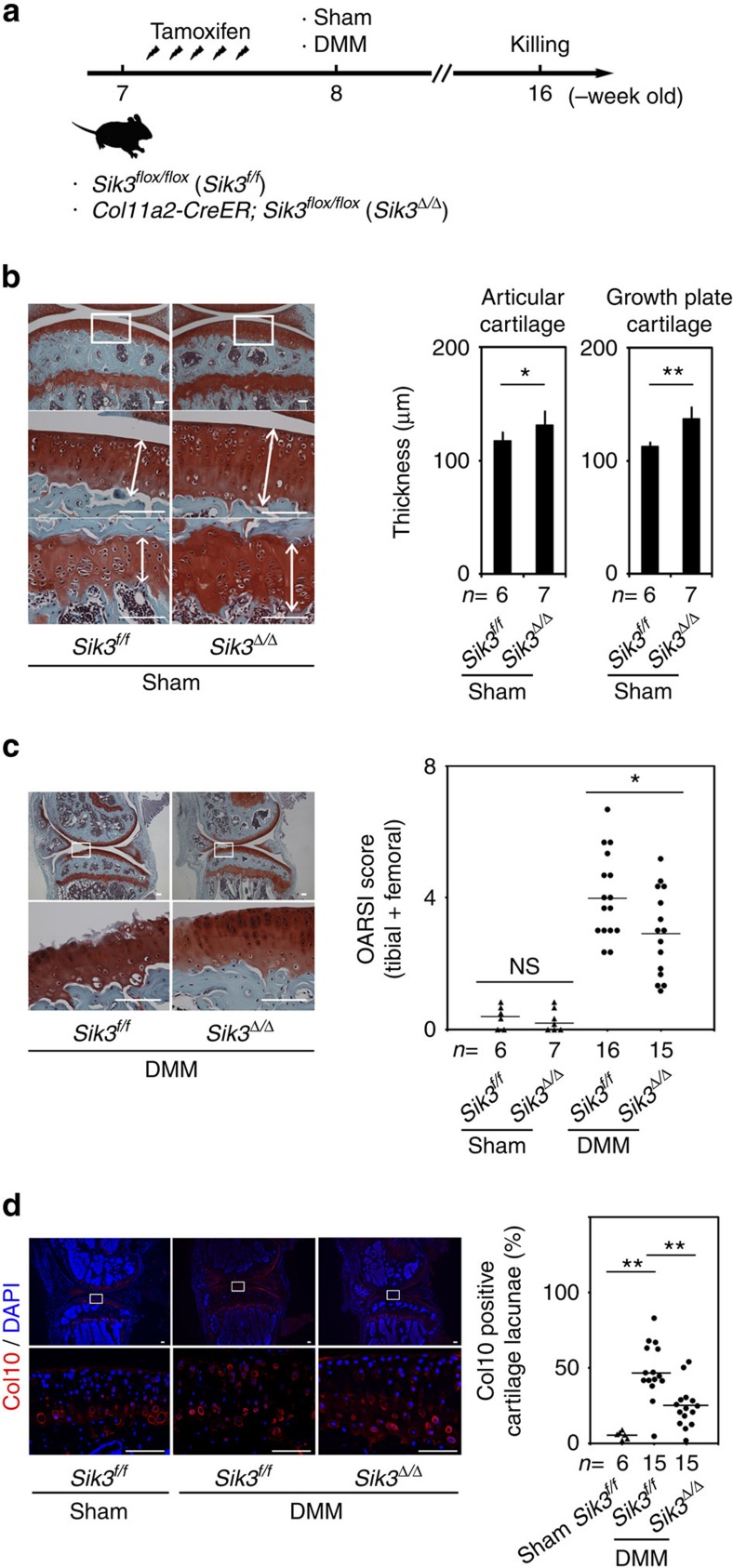
Effects of Sik3 deletion on osteoarthritis development in DMM-operated knees. (**a**) Protocol of experiments: 7-week-old mice were intraperitoneally injected with tamoxifen for 5 consecutive days, had their right knees subjected to DMM or sham surgeries, and were killed at 16 weeks of age. (**b**) Histology of proximal tibia in sham-operated knees. Safranin O-fast green-iron haematoxylin staining. The thickness of articular cartilage and growth plate cartilage are shown in the graph. *t*-test. (**c**) Histology of articular cartilage of proximal tibia in DMM-operated knees. Boxed regions are shown in the bottom panels. OARSI scores of articular cartilage in sham- and DMM-operated knees are shown. Two blinded scorers gave consistent results. Mann–Whitney test. (**d**) Immunohistochemistry for Col10 in knees. Boxed regions in the top panels are shown in the bottom panels. The percentage of lacunae surrounded by Col10-positive matrix per total number of lacunae are shown in the right graph. Tukey–Kramer *post hoc* test. Error bars denote means±s.d. Numbers of mice examined are indicated at the bottom of the graphs. *P<0.05 and **P<0.01. NS, not significantly different. Scale bars, 100 μm.

We next examined whether Sik3 deletion affected the development of osteoarthritis in adult mice. We treated 7-week-old mice with tamoxifen to prepare *Sik3*^*Δ/Δ*^ and *Sik3*^*f/f*^ mice, and subsequently subjected their right knees to destabilized medial meniscus (DMM) surgery or sham surgery (skin and joint capsule incision) at 8 weeks of age. The mice were killed 8 weeks after the operation (therefore at 16 weeks of age) and subjected to histological analysis (Fig. 2a). The thickness of articular cartilage in sham-operated knees in *Sik3*^*Δ/Δ*^ mice was thicker than that in *Sik3*^*f/f*^ mice (Fig. 2b), confirming that Sik3 deletion caused thickening of articular cartilage in adult mice. DMM operation caused less severe osteoarthritis in *Sik3*^*Δ/Δ*^ mice than in *Sik3*^*f/f*^ mice (Fig. 2c). Immunohistochemical analysis showed that DMM operation induced Col10 expression in the non-calcified zone of articular cartilage of *Sik3*^*f/f*^ mice (Fig. 2d). Deletion of Sik3 reduced this induction, as indicated by the limited expression of Col10 in the *Sik3*^*Δ/Δ*^ mouse knee joint that received the DMM operation. These results collectively suggest that deletion of Sik3 in chondrocytes thickens articular cartilage and inhibits activation of the hypertrophy programme to protect mice from developing osteoarthritis.

We further examined whether Sik3 deletion in chondrocytes affects extra-cartilage status, which could affect cartilage biology and pathology. Body weights and locomotor activities indicated by a rotarod performance test were not different between *Sik3*^*flox/flox*^ and *Col11a2-CreER; Sik3*^*flox/flox*^ mice before or 8 weeks after administration of tamoxifen (Supplementary Fig. 4b). Angiogenesis indicated by the amount of CD31-positive endothelial progenitor cells in subchondral bone was also not different between *Sik3*^*flox/flox*^ and *Col11a2-CreER; Sik3*^*flox/flox*^ mice after administration of tamoxifen (Supplementary Fig. 4c). These results suggest that *Sik3* deletion in cartilage affected cartilage biology and pathology autonomously.

### Identification of pterosin B as a Sik3 inhibitor

**Figure 3:**
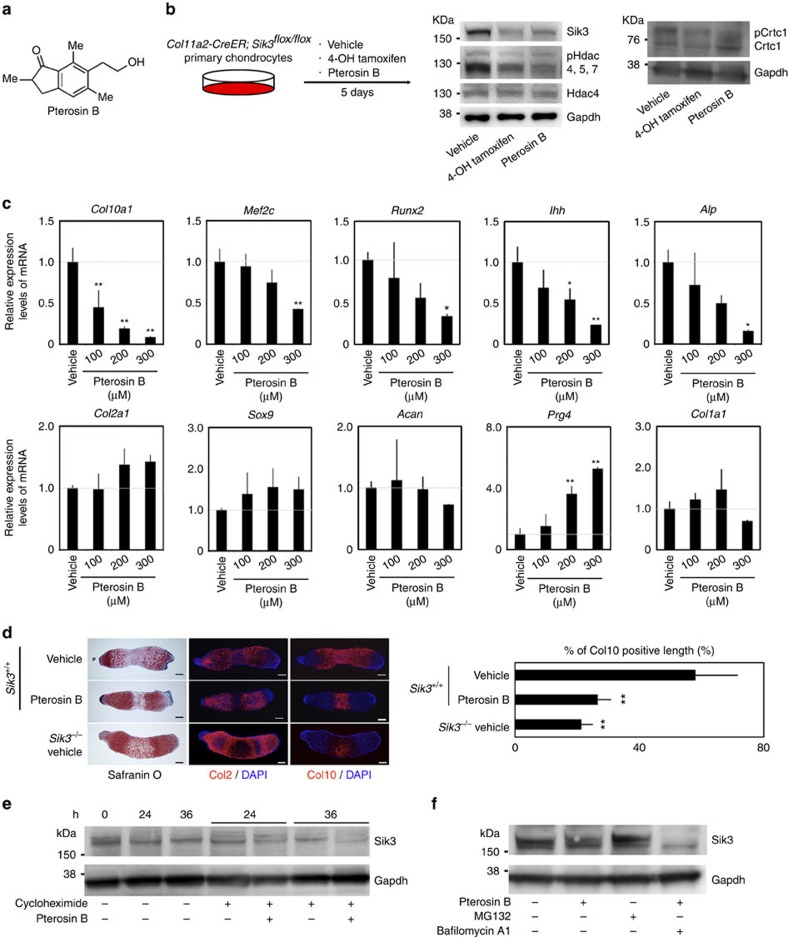
Effects of pterosin B on chondrocyte differentiation *in vitro*. (**a**) Structure of pterosin B. (**b**) Primary chondrocytes prepared from *Col11a2-CreER; Sik3*^*flox/flox*^ mice were treated with vehicle, 0.5 μM 4-OH tamoxifen or 300 μM pterosin B for 5 days and subjected to western blot analysis. The images are representative of two independent experiments. (**c**) Real-time RT–PCR expression analysis of marker genes in pellet culture of mouse primary chondrocytes with pterosin B; *n*=3 pellets. (**d**) Metatarsal primordial cartilage from *Sik3*^*+/+*^ mice were organ-cultured in 300 μM pterosin B or vehicle. Metatarsal primordial cartilage from conventional Sik3 knockout mice were used for the control. Semiserial histological sections were stained with safranin O-fast green-iron haematoxylin and immunostained for Col2 and Col10. The percentage of Col10-postive area per total length in the axial direction is indicated; *n*=3 metatarsals. (**e**) Primary chondrocytes were treated with 100 μg ml^−1^ cycloheximide in the presence or absence of 300 μM pterosin B. Cells were lysed after treatment for 0, 24 and 36 h, and cell lysates were subjected to western blot analysis for Sik3 and Gapdh. The images are representative of two independent experiments. (**f**) Primary chondrocytes were incubated in the presence or absence of 1 μM MG132, 10 nM bafilomycin A1 or 300 μM pterosin B for 36 h. Cell lysates were subjected to western blot analysis for Sik3 and Gapdh. The images are representative of two independent experiments. Error bars denote the means±s.d. *P<0.05 and **P<0.01 by the Tukey–Kramer *post hoc* test. Scale bars, 100 μm.

Because our *Sik3*^*Δ/Δ*^ mice data suggested that Sik3 could be a target for treatment of osteoarthritis, we searched for compounds that inhibit Sik3 activity. It is known that SIKs phosphorylate HDACs and CREB-regulated transcription coactivators (CRTCs) to translocate these molecules from the nucleus to cytoplasm, which thus abolishes their function in the nucleus. HDAC inhibits myocyte enhancer factor 2C (MEF2C), and CRTC activates CREB in nuclei. We established a dual luciferase assay system to monitor MEF2C and CRTC2 activities in HEK293 cells. We screened ∼2,500 compounds and found that pterosin B (Fig. 3a) decreased MEF2C activity and increased CRTC2 activity^14^. Pterosin B is an extract acquired from boiling *Pteridium aquilinum* in water supplemented with sodium bicarbonate. We then developed a method to synthesize pterosin B (Supplementary Fig. 5a). Our synthesized pterosin B inhibited MEF2C and activated CRTC2 in a dose-dependent manner in HEK293 cells according to a luciferase assay system in which luciferase activity was regulated by GAL4-fusion MEF2 or GAL4-fusion CRTC2 (Supplementary Fig. 5b). These results confirmed that pterosin B and not some impurity in the extract was responsible for the function. Further, we confirmed that pterosin B specifically inhibited Sik3 in ATDC5 cells (Supplementary Fig. 5c). Thus, we used synthesized pterosin B in the following experiments.

Western blot analysis showed that the addition of pterosin B decreased the amounts of Sik3, phosphorylated Hdac4 and phosphorylated Crtc1 in primary chondrocytes in a manner similar to 4-OH tamoxifen in chondrocytes from *Col11a2-CreER; Sik3*^*flox/flox*^ mice (Fig. 3b).

Sik3 accelerates chondrocyte hypertrophy by anchoring Hdac4 to the cytoplasm and releasing Mef2c from inhibition by Hdac4 in the nucleus^10^. Mef2c accelerates the hypertrophy programme in chondrocytes^15^. We examined whether pterosin B inhibits hypertrophic differentiation of chondrocytes by performing pellet culture of wild-type mouse primary chondrocytes in the presence or absence of pterosin B for 4 weeks. The presence of pterosin B decreased the expression levels of *Col10*, *Mef2c* and *Alp* in a dose-dependent manner (Fig. 3c), similar to how tamoxifen decreased these levels in pellet culture of *Col11a2-CreER; Sik3*^*flox/flox*^ chondrocytes (Supplementary Fig. 3c). It also significantly decreased *Runx2* and *Ihh* expression levels (Fig. 3c), suggesting that pterosin B inhibits prehypertrophy of chondrocytes as well. On the other hand, we did not detect significant changes in the expression levels of *Sox9*, *Col2a1* or *Acan* mRNAs in pellets with pterosin B treatment (Fig. 3c), which resembles tamoxifen effects in pellet culture of *Col11a2-CreER; Sik3*^*flox/flox*^ chondrocytes (Supplementary Fig. 3c). We suspect the expression levels of these three factors did not increase when hypertrophy was inhibited by Sik3 deletion or pterosin B, because the pellet culture samples were mainly composed of *Col2a1*-expressing chondrocytes and contained a limited population of hypertrophic chondrocytes. Finally, pterosin B did not significantly affect the expression of catabolic enzymes, such as *Mmp3*, *Mmp13*, *Adamts4* or *Adamts5* (Supplementary Fig. 6a).

In addition, pterosin B increased the expression level of *Prg4* (Fig. 3c), which encodes lubricin, in wild-type chondrocyte pellets. A CBP-CREB interaction inhibitor partly abolished this elevation of *Prg4* expression, suggesting that pterosin B treatment activates *Prg4* expression, at least in part, through the CRTC/CREB pathway (Supplementary Fig. 6b). We also confirmed that pterosin B inhibits chondrocyte hypertrophy in organ culture of metatarsal primordial cartilage (Fig. 3d), as it reduced the area of the Col10-positive region in organ-cultured primordial cartilage of wild-type mice.

RNA-sequencing analysis of entire mRNA transcripts showed that chondrocyte pellets cultured in the presence or absence of pterosin B and cartilage tissues were clustered distinctly from other tissues such as skin, bone, muscle, liver, kidney, small intestine, testis, brain and spinal cord (Supplementary Fig. 7). Real-time PCR with reverse transcription (RT–PCR) expression analysis showed pterosin B did not significantly change *Col1a1* expression levels (Fig. 3c). These results suggest that pterosin B regulates the differentiation of chondrocytes, but does not alter the cellular phenotype to other types of cells. RNA sequencing analysis (Supplementary Fig. 8) largely confirmed the changes in the expression of marker genes found by real-time RT–PCR analysis (Fig. 3c). Gene Ontology term analysis demonstrated that downregulated genes in pterosin B-treated cells were associated with bone mineralization (Supplementary Table 1), agreeing with the observation that pterosin B delays chondrocyte hypertrophy and mineralization. In addition, KEGG (Kyoto Encyclopedia of Genes and Genomes) pathway analysis indicated that hedgehog signalling was attenuated by pterosin B. The inhibition of hedgehog signalling attenuates the severity of osteoarthritis^6^. Whether hedgehog signalling mediates effects of Sik3 on osteoarthritis is unexplored.

We finally analysed how pterosin B affects SIK3 activity. Western blot analysis showed that treatment with pterosin B decreased the amount of Sik3 protein (Fig. 3b). This decrease was accelerated in cells treated with cycloheximide, an inhibitor of protein synthesis (Fig. 3e). We also found that MG132 increased the amount of Sik3 protein, whereas bafilomycin A1 did not (Fig. 3f). Combined, these results suggest that pterosin B induces the degradation of Sik3 protein by a proteasomal pathway. SIK3 is activated by phosphorylation at T163 (ref. 16) and inactivated by phosphorylation at T411 or S493 (ref. 17). We recently found that pterosin B upregulates the phosphorylation of SIK3 at T411 and S493 in hepatocytes, which results in the suppression of SIK3 activity^14^. Whether phosphorylation at T411 or S493 is related to SIK3 degradation in chondrocytes is for further study.

### Pterosin B protects cartilage from osteoarthritis

**Figure 4:**
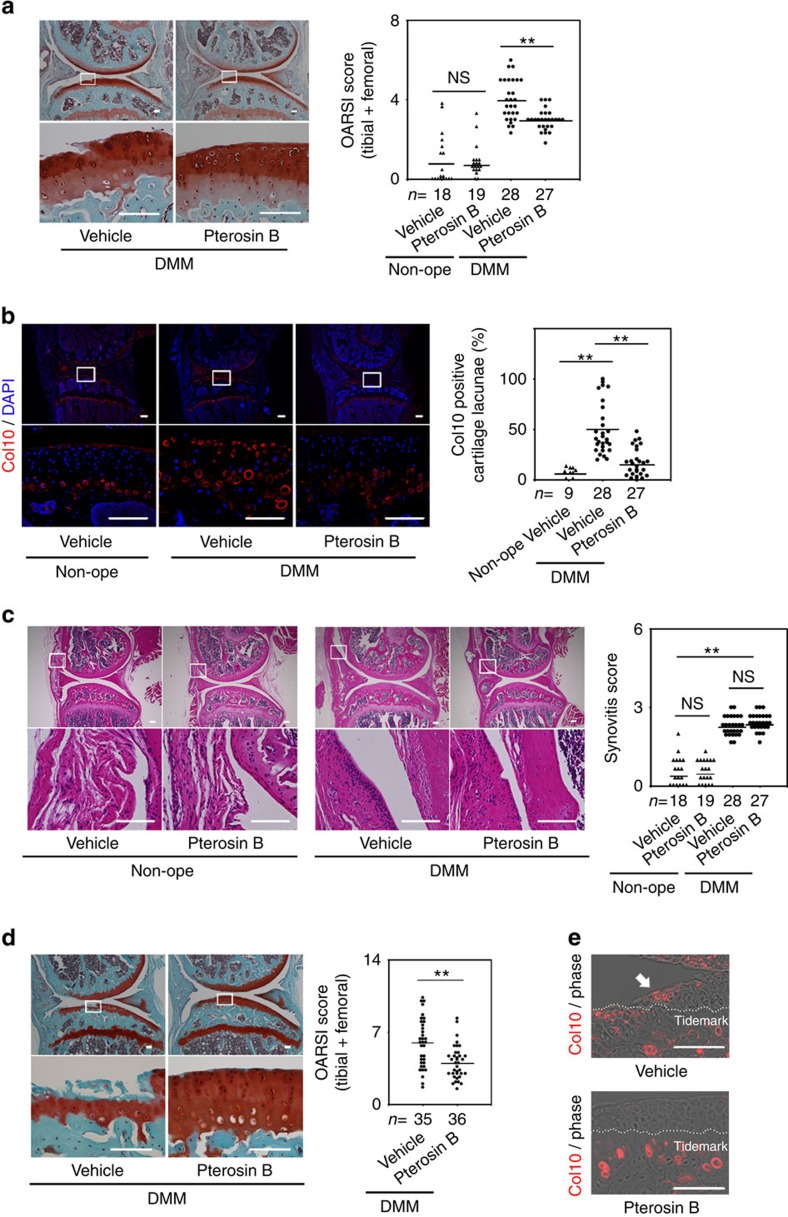
Effects of pterosin B on osteoarthritis development in DMM-operated knees. (**a**–**c**) Eight-week-old mice with DMM were treated with pterosin B for 8 weeks. (**a**) Histology of articular cartilage of DMM-operated knees injected with pterosin B or vehicle. Safranin O-fast green-iron haematoxylin staining. Boxed regions in the top panels are shown in the bottom panels. OARSI scores of articular cartilage in DMM-operated knees injected with pterosin B or vehicle (black dots) are shown to the right. OARSI scoring was also done on non-operated knees injected with pterosin B or vehicle (black triangles). Two blinded scorers gave consistent results. Representative data are shown; Mann–Whitney test. (**b**) Immunohistochemistry for Col10 in knees. Boxed regions in the top panels are shown in the bottom panels. The percentage of lacunae surrounded by Col10-positive matrix per total number of lacunae is indicated. Tukey–Kramer *post hoc* test. (**c**) Histology of synovium; haematoxylin–eosin staining. Boxed regions in the top panels are shown in the bottom panels. Synovitis scores in DMM-operated knees injected with pterosin B or vehicle (black dots) and in non-operated knees injected with pterosin B or vehicle (black triangles); Mann–Whitney test. (**d**,**e**) Thirteen-week-old mice with DMM were treated with pterosin B for 12 weeks. (**d**) Histology of articular cartilage of DMM-operated knees injected with pterosin B or vehicle. Safranin O-fast green-iron haematoxylin staining. Boxed regions in the top panels are shown in the bottom panels. OARSI scores of articular cartilage in DMM-operated knees injected with pterosin B or vehicle are shown to the right. Two blinded scorers gave consistent results. Representative data are shown; Mann–Whitney test. (**e**) Immunohistochemistry for Col10 in articular cartilage in DMM-operated knees injected with pterosin B or vehicle. White dotted lines indicate tidemarks. White arrow represents Col10 expression above the tidemark. The images are representative of two independent experiments. Number of mice examined is indicated at the bottom of the graphs. *P<0.05 and **P<0.01. NS, not significantly different. Scale bars, 100 μm.

We examined whether pterosin B can control osteoarthritis development. We performed DMM surgery on the right knees of 8-week-old wild-type mice (Fig. 4a). We injected pterosin B or vehicle solutions into the joint space of DMM-operated knees three times a week for 8 weeks. We also injected the right knees of mice, which were not subjected to operation with pterosin B or vehicle using the same schedule. We killed the mice at 16 weeks of age. OARSI scores were better in the pterosin B-treated knees with DMM than vehicle-treated knees with DMM. The non-operated knees treated with pterosin B looked normal and showed similar OARSI scores with those of non-operated knees treated with vehicle. These results suggest that pterosin B protects mice from osteoarthritis development. Immunohistochemical analysis showed that pterosin B treatment reduced the Col10 expression activated by DMM in the non-calcified zone of articular cartilage (Fig. 4b). Pterosin B treatment did not significantly change the synovitis score in non-operated knees or DMM-operated knees (Fig. 4c).

We also examined the effects of pterosin B on older mice. We performed DMM surgery at 13 weeks of age and injected pterosin B or vehicle solutions into the joint space of the DMM-operated knees three times a week for 12 weeks (Fig. 4d). Again, OARSI scores were better in the pterosin B-treated knees with DMM than vehicle-treated knees with DMM (Fig. 4d). Pterosin B treatment reduced Col10 expression in the non-calcified zone of articular cartilage DMM-operated knees (Fig. 4e).

### Pterosin B inhibits hypertrophy of human chondrocytes

**Figure 5:**
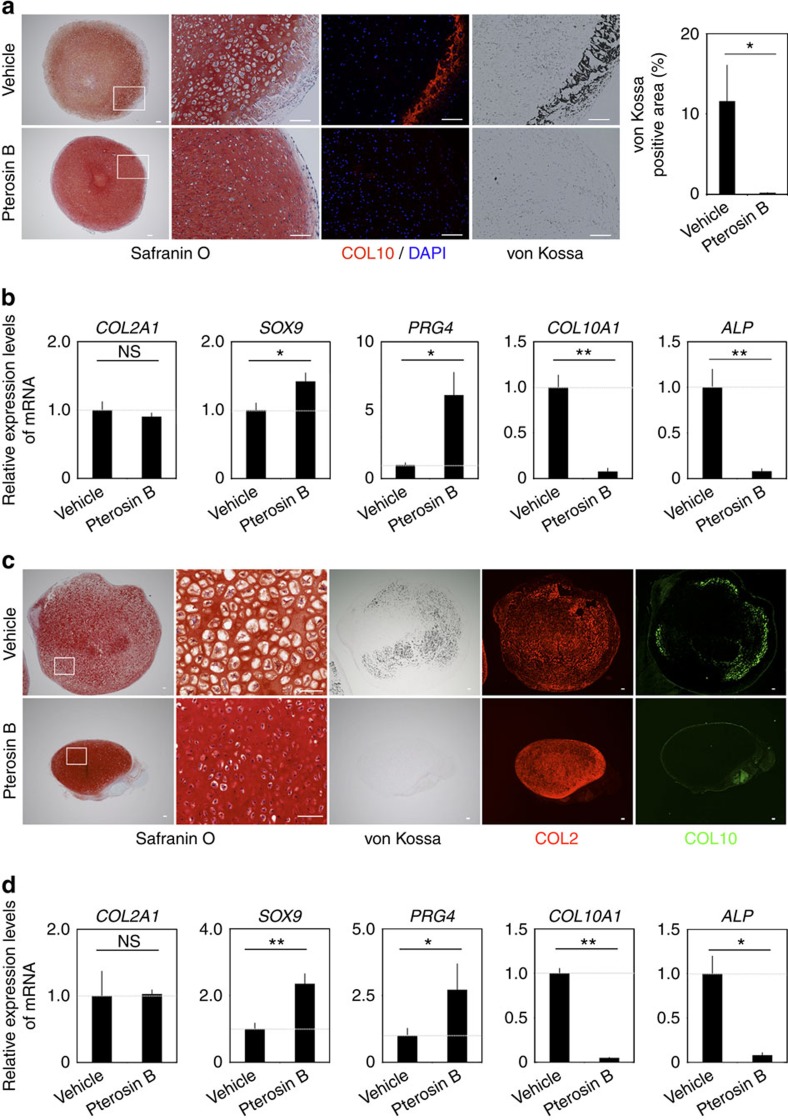
Effects of pterosin B on human articular chondrocytes and hiPSC-derived chondrocytes. (**a**) Semiserial histological sections of cultured pellets of human articular cartilage in the presence or absence of 300 μM pterosin B were stained with safranin O-fast green-iron haematoxylin and von Kossa and immunostained for COL10. Boxed regions in the left panels are shown in the right. Area of von Kossa-positive regions per total area of pellets are shown; *n*=3 pellets. (**b**) Real-time RT–PCR expression analysis of marker genes in pellet culture of human articular chondrocytes in the presence or absence of 300 μM pterosin B; *n*=3 pellets. (**c**) Semiserial histological sections of hiPSC-derived cartilage particles treated with or without 300 μM pterosin B were stained with safranin O-fast green-iron haematoxylin and von Kossa and immunostained for COL2 and COL10. Boxed regions in the left panels are shown in the right. The images are representative of three pellets each. (**d**) Real-time RT–PCR expression analysis of marker genes in hiPSC-derived cartilage particles in the presence or absence of 300 μM pterosin B; *n*=3 pellets. Error bars denote means±s.d. *P<0.05, **P<0.01 and NS, not significantly different by the *t*-test. Scale bars, 100 μm.

We finally examined whether pterosin B inhibits hypertrophy of human articular chondrocytes. Human articular chondrocytes were subjected to pellets cultured in chondrogenic medium for 8 weeks. We further cultured the chondrocyte pellets in medium containing BMP-2, GDF-5, 3,3,5-Triiodo-L-thyronine (T3) and β-glycerophosphate for 2 weeks to induce hypertrophic differentiation. Histological analysis of the pellets revealed that the addition of pterosin B in the medium protected chondrocytes from hypertrophy and mineralization (Fig. 5a). Real-time RT–PCR analysis showed that pterosin B reduced the expression levels of *COL10A1* and *ALP*, did not change that of *COL2A1* and increased those of *SOX9* and *PRG4* (Fig. 5b).

We next examined the effects of pterosin B on chondrocytes derived from human-induced pluripotent stem cells (hiPSCs). We differentiated hiPSCs towards chondroyctes and subjected them to suspension culture to produce cartilage particles for 8 weeks, as we described previously^18^. The cartilage particles were further cultured with pterosin B or vehicle in hypertrophic condition for 4 weeks. Pterosin B protected chondrocytes in the particles from hypertrophy and mineralization, as indicated by histological appearance, von Kossa staining, immunohistochemical staining for COL10 (Fig. 5c) and the expression of marker genes (Fig. 5d). These results collectively suggest that pterosin B inhibits hypertrophic change of human articular chondrocytes and hiPSC-derived chondrocytes.

## Discussion

Here we show that genetic deletion of *Sik3* or inhibition of the Sik3 signal by pterosin B protected mouse cartilage from osteoarthritic change caused by DMM. The mechanisms of the protection appear as inhibition of activation of the hypertrophy programme or elevation of the *Prg4* expression level in articular chondrocytes.

Although definite evidence is lacking, age-associated thinning of articular cartilage is a suspect risk factor for the erosion of articular cartilage in osteoarthritis^3^. The turnover of articular cartilage, which should define the thickness of articular cartilage, is not well understood. The developmental process of the secondary ossification centre raises the hypothesis that cells in the non-calcified zone very slowly turn into cells that express Col10 and reside in the calcified zone, where they are subsequently removed and replaced by subchondral bone. A recent study using lineage tracing of *Prg4*-positive cells has shown that cells in the deeper zone are the progeny of cells in the non-calcified zone^19^, supporting this hypothesis. The thickness of articular cartilage decreases with age, probably due to an increasing imbalance between the proliferation rate of non-calcified zone cells and the rate by which these cells turn into cells that express Col10, reside in the calcified zone and are finally removed. In this study, we found that Sik3 deletion increased the population of chondrocytes and thickened articular cartilage at least in young mice. Examining the effects of Sik3 deletion on age-associated osteoarthritis model in mice is for further study.

Independent research groups have confirmed two types of substrates for SIKs, CRTC (a coactivator for the cAMP response element (CRE)-binding protein (CREB)] and class IIa histone deacetylase (HDAC)^20^. SIKs phosphorylate these two substrates to induce nuclear export, resulting in a loss of the substrates' transcriptional regulatory activities^21^,^22^. Sik3 anchors Hdac4 in the cytoplasm to release Mef2c from inhibition by Hdac4, thus activating the hypertrophy programme in the nuclei of growth plate cartilage^10^,^15^,^23^. It has been reported that HDAC4 is decreased in osteoarthritis cartilage, which may contribute to the pathogenesis of osteoarthritis cartilage degeneration^24^. These findings are consistent with our observation that Sik3 deletion or pterosin B administration protected cartilage from osteoarthritic change through activation of Hdac4, which prevented Mef2c from activating the hypertrophy programme in articular chondrocytes.

Prg4 is secreted by the most superficial articular chondrocytes and contributes to the lubrication of joints^25^. It also protects cartilage against osteoarthritic change^26^,^27^, and its expression is activated by the CRTC/CREB complex in articular chondrocytes^28^. Sik3 anchors CRTC2 in the cytoplasm, preventing CRTC2 from co-activating CREB in nuclei^29^. We found that pterosin B treatment increased the expression level of *Prg4* in chondrocytes, at least in part, through CREB activation. These findings collectively suggest that Sik3 deletion or pterosin B administration protect cartilage from osteoarthritic change through the CRTC/CREB pathway to activate *Prg4* expression.

There are some limitations in this study. First, we cannot exclude the possibility that thickening of growth plates affected the course of osteoarthritis development in *Sik3*^*Δ/Δ*^ mice. Second, DMM surgery was performed on relatively young *Sik3*^*Δ/Δ*^ mice. An analysis of the effects of Sik3 deletion on aged mice with DMM surgery would further clarify whether Sik3 affects the development of osteoarthritis, since osteoarthritis is more common in the elderly. Third, because we are not sure whether injections of pterosin B were always done intraarticularly and because pterosin B should be absorbed in the joint, we cannot dismiss extraarticular or systemic effects. Fourth and finally, other effects of pterosin B besides Sik3 inhibition cannot be ruled out as providing protection from osteoarthritis. Nevertheless, our study shows that SIK3 was activated in human osteoarthritic chondrocytes and that pterosin B inhibited hypertrophic changes and increased *PRG4* expression in human articular chondrocytes and hiPSC-derived chondrocytes. Pterosin B is a compound in a cooked plant consumed in human diets. On the basis of our results, we suggest that SIK3 regulates the homeostasis of articular cartilage and can be a target for the treatment of osteoarthritis, and that pterosin B can be the lead compound for relevant drugs.

## Methods

### Ethics statement

All the experiments were approved by the institutional review board, institutional animal committee (as appropriate) and the institutional biosafety committee of Kyoto University, and the institutional review board of the University of Toyama and Hachiya Orthopaedic Hospital.

### Generation of mice

A cDNA encoding the modified estrogen receptor ligand binding domain (ER) (provided by Dr M. Iwamoto, Children's Hospital of Philadelphia)^30^ was linked to the *Cre* sequence to create the *CreER* gene. The *lacZ* gene in 742*LacZ*Int^31^ was replaced with the CreER gene to create *Col11a2-CreER*, and transgenic mice bearing the *Col11a2-CreER* were generated.

C57BL/6N ES cell clones, which contained the *Sik3*^*tm1a (EUCOMM) Hmgu*^ allele, were purchased from the European Conditional Mouse Mutagenesis Program (EUCOMM). This allele includes targeting cassettes that were flanked by FRT recombination sites to allow removal by Flp recombinase and a pair of loxP recombination sites around exon 5 of *Sik3* (Supplementary Fig. 3a). These targeted ES cells were injected into blastocysts of Slc:ICR mice to generate ES cell-derived chimeras. Male chimeras were mated with C57BL/6NCrSlc female mice, and germ line transmission was assessed by PCR. Heterozygous F1 mice were mated with B6-Tg(CAG-FLPe)36 mice, which were provided by the RIKEN BRC through the National Bio-Resource Project of MEXT, Japan, to delete the Neo and LacZ cassettes and generate a floxed allele (*Sik3*^*flox/+*^). Cre recombinases can induce gene inactivation of the *Sik3*^*flox*^ allele by deleting exon 5 to generate a frame shift.

To generate *Sik3* conditional knockout mice, *Col11a2-CreER* transgenic mice and *Sik3*^*flox/flox*^ mice were mated. Then, *Col11a2-CreER; Sik3*^*flox/flox*^ and *Rosa26-stop*^*flox*^*-EYFP* mice (Jackson Laboratory) were intercrossed and *Col11a2-CreER; Sik3*^*flox/flox*^*; Rosa26-stop*^*flox*^*-EYFP* mice were generated. *Col11a2-CreER; Sik3*^*flox/+*^; *Rosa26-stop*^*flox*^*-EYFP* mice were used as controls.

### Human articular cartilage samples and OARSI grading

Articular cartilages were collected from 16 patients suffering from medial type knee osteoarthritis at the time of total knee arthroplasty. A cartilage fragment was dissected from each individual and subjected to histological analysis. Normal articular cartilage samples were prepared from cadavers who had been free from joint disorders. The age and gender of the individuals are shown in Supplementary Tables 2 and 3. Semiserial sections were used for OARSI grading and immunostained for SIK3 and pSIK3. OARSI grading was performed by one individual^13^. Use of human materials has been approved by the institutional review board of Kyoto University, University of Toyama and Hachiya Orthopaedic Hospital.

### Histological analysis

For frozen sections, the samples were harvested without fixation and were immediately embedded in SCEM compound (SECTION-LAB). The sections were prepared at 6-μm thickness with a Cryofilm type 2c(9) (SECTION-LAB) using a CM3050S cryomicrotome (Leica) according to the method described by Kawamoto^32^.

For paraffin-embedded sections, the samples were dissected, fixed in 4% paraformaldehyde, processed and embedded in paraffin. The sections were prepared at 6-μm thickness.

One individual measured the cartilage thickness and cell number of the histological sections in a blinded manner.

### Introduction of osteoarthritis model into *Sik3* ^*Δ/Δ*^ mice

A 100 μg per gram of body weight tamoxifen (Sigma) was intraperitoneally injected into the peritoneal spaces of 7-week-old *Col11a2-CreER; Sik3*^*flox/flox*^ and *Sik3*^*flox/flox*^ male mice daily for 5 consecutive days. Then, the right knee joints of the mice were subjected to DMM surgery^33^ or sham surgery (skin and joint capsule incision) at 8 weeks of age. The mice were killed 8 weeks after the operation, and the knee joints were subjected to histological analysis.

### Rotarod performance test

The mice were put on a Rota-Rod (UGO BASILE). The speed of the rotor drums were increased from 4 to 78 r.p.m. at 0.25 r.p.m. s^−1^ acceleration. The latency to fall from the rotor drums was recorded. The results from three trials were averaged.

### Preparation of reagents

A 100 mg tamoxifen was dissolved in 2 ml ethanol and then suspended in 8 ml corn oil. 4-OH tamoxifen (Sigma) was dissolved in ethanol at a concentration of 0.5 mM to prepare stock solution. Cyclophosphamide (Sigma) and bafilomycin A1 (Sigma) were dissolved in DMSO at a concentration of 100 mg ml^−1^ and 100 μM, respectively, to prepare stock solution. CBP-CREB interaction inhibitor (Cas 92–78–4, Merck Millipore) was also diluted in DMSO at a concentration of 20 mM as stock solution. Recombinant mouse IL-1β/IL-1F2 (R&D systems) were prepared at a concentration of 10 μg ml^−1^ in sterile PBS with 0.1% bovine serum albumin. Ten millimolar ready to use MG132 solution (Sigma) was purchased. Pterosin B was synthesized by Intelium Corp. (Supplementary Fig. 5a). Pterosin B was dissolved in DMSO at a concentration of 100, 200 and 300 mM for *in vitro* experiments and 900 mM for mouse experiments to prepare stock solution. These solutions were diluted with culture medium for *in vitro* experiments.

### Application of pterosin B to osteoarthritis model mice

DMM operations were performed on the right knee joints of C57BL/6NCrSlc male mice 8 weeks or 13 weeks of age. Nine hundred millimolar pterosin B stock solution was diluted with PBS to prepare the injection solution. Fifteen microlitres either of 900 μM pterosin B injection solution or vehicle was injected into the intraarticular spaces of the knee joints threetimes a week. After 8 or 13 weeks of injection, mice were killed, and the knee joints were subjected to histological analysis.

### OARSI scoring of articular cartilage of mouse knee joints

Twelve paraffin-embedded semiserial sagittal sections with 40 μm intervals were prepared from the medial compartment of each mouse knee joint. Sections were stained with safranin O-fast Green-iron haematoxylin. Cartilage destruction was scored using the OARSI scoring system^34^. OARSI scoring was performed by two independent scorers in a blinded manner. Six consecutive sections out of 12 were selected to represent the weight-bearing area of the femur and tibia, respectively, and were subjected to OARSI scoring. The worst three scores were selected, and their average were calculated. The scores of the femur and that of tibia were summed and designated as the OARSI score for each knee. Synovial inflammation was scored as described previously^35^.

### Immunofluorescent staining of histological sections

Frozen sections were fixed with 4% paraformaldehyde for 5 min. After washing with TBST, the sections were incubated with 10 mg ml^−1^ hyaluronidase (Sigma) at 37 °C for 30 min. After blocking with 10% goat normal serum (Nichirei Corporation), the sections were incubated with primary antibody for 2 h at room temperature.

Paraffin-embedded sections were deparaffinized and incubated in 1 mM EDTA (pH 8.0) at 80 °C for 15 min to retrieve the antigen. Then, sections were treated with 10 mg ml^−1^ hyaluronidase at 37 °C for 30 min. For detection of type X collagen, the samples were treated with 20 μg ml^−1^ Proteinase K (Life Technologies) for 5 min. After blocking with 10% goat normal serum, the sections were incubated with primary antibody for 2 h at room temperature.

The primary antibodies used were as follows: mouse anti-type II collagen (Thermo Scientific, 1:500), rabbit anti-type X collagen (Cosmo bio, 1:600), rabbit anti-SOX9 (Santa Cruz Biotechnology, 1:200), anti-CD31 (Abcam, 1:20) and chicken anti-GFP (for recognition of EYFP) (Abcam, 1:800). The anti-pSIK3(pT163) antibodies were raised in rabbit against peptides corresponding to residues (CSNLFTPGQLLK(pT)W, pT: phospho-Thr) of human SIK3 (Sigma, 1:2,000). Immune complexes were detected using secondary antibodies conjugated to Alexa Fluor (Life Technologies, 1:1,000). DAPI (Dojindo Molecular Technologies, 1:1,000) was used.

### BrdU staining

The mice were intraperitoneally injected with BrdU labelling reagent (10 μl g^−1^ body weight, Life Technologies) 3 h before being killed. The paraffin-embedded sections were prepared. The incorporated BrdU was detected using a BrdU staining kit (Life Technologies) to distinguish actively proliferating cells.

### Pellet culture of mouse primary chondrocytes

Primary chondrocytes were prepared from newborn mice as described previously^36^. Briefly, epiphyseal cartilage was dissected from the knee joints and femoral heads of newborn mice, predigested with 3 mg ml^−1^ collagenase D (Roche) in Dulbecco's Modified Eagle Medium (DMEM) (Sigma) at 37 °C for 90 min and further digested in 0.5 mg ml^−1^ collagenase D at 37 °C overnight. A total of 5 × 10^5^ primary chondrocytes were plated in a 10-cm dish and cultured in DMEM, 10% FBS, 2 mM L-Glu (Life Technologies), 1% penicillin–streptomycin (Life Technologies) for 10 days. A total 0.5 μM 4-OH tamoxifen was added for the first 5 days. Then, 5 × 10^5^ primary chondrocytes were transferred into a 15-ml tube (Thermo Fisher Scientific) and centrifuged at 500 g for 10 min. After 3 days incubation in the same medium in the tubes, the resulting cell pellets were transferred into petri dishes and incubated in the same medium in the presence or absence of various concentrations of pterosin B for a further 4 weeks.

### Organ culture of mouse metatarsal primordial cartilage

Second, third and fourth metatarsal primordial cartilage of forelimb bud was cultured as described previously^37^. Metatarsal cartilage was dissected from mouse embryos at 15.5 days postcoitum. One metatarsal was organ-cultured in each well. From the next day, the metatarsals were treated with 300 μM pterosin B or vehicle for 1 week. Metatarsals were subjected to histological analysis.

### Pellet culture of human articular chondrocytes

Cartilage fragments were dissected from the less damaged area of the articular surface in the posterior condyle of the distal femur that was discarded at the time of total knee replacement surgery. The cartilage fragments were minced and treated with 0.25% trypsin at 37 °C for 1 h. Cartilage fragments were further digested in 4 mg ml^−1^ collagenase D at 37 °C overnight. A total of 1 × 10^6^ human articular chondrocytes were cultured in a 10-cm dish in chondrogenic medium, which consisted of DMEM, 10% FBS, 1% insulin–transferrin–selenium solution (ITS, Life Technologies), 10 ng ml^−1^ recombinant human TGF-β1 (Peprotech), 100 nM dexamethasone (Sigma), 50 μg ml^−1^ L(+)-ascorbic acid (Nacalai tesque), 1 mM sodium pyruvate (Life Technologies) and 1% penicillin–streptomycin for 7 days. Then, 5 × 10^5^ primary human chondrocytes were transferred into a 15-ml tube and centrifuged at 500 g for 10 min. After 3 days incubation in the same medium in the tubes, the resulting cell pellets were transferred into a petri dish and incubated in the same medium for a further 8 weeks. Finally, the medium was replaced with hypertrophic medium (DMEM supplemented with 1% FBS, 1% ITS, 100 ng ml^−1^ BMP-2 (Peprotech), 10 ng ml^−1^ GDF-5 (PTT), 1 μM 3,3,5-Triiodo-L-thyronine (T3) (Sigma), 50 μg ml^−1^ L(+)-Ascorbic acid, 10 nM dexamethasone, 10 mM β-glycerophosphate (Sigma) and 1% penicillin–streptomycin) in the presence or absence of 300 μM pterosin B for an additional 2 weeks.

### Hypertrophic differentiation of human iPSC-derived cartilage

Human iPSCs (hiPSCs) were chondrogenically differentiated to produce hiPSC-derived cartilaginous particles as described previously^38^. The hiPSCs were transferred and then maintained in a feeder-free medium, Essential 8 (Invitrogen) with 50 units ml^−1^ penicillin and 50 mg ml streptomycin, in 3.5-cm Matrigel-coated dishes. The hiPSCs formed high-density cell colonies which consisted of 1–2 × 10^5^ cells 10–15 days after the start of maintenance under the feeder-free culture conditions. Subsequently, the chondrogenic differentiation of iPSCs was performed. The hiPSCs were initially differentiated into mesendodermal cells in DMEM/F12 (Sigma) with 10 ng ml^−1^ of Wnt3A (R&D), 10 ng ml^−1^ of Activin A (R&D), 1% ITS, 1% FBS and 50 units and 50 mg ml^−1^ of penicillin and streptomycin, respectively for 3 days. On day 3, the medium was changed to the basal medium (DMEM with 1% ITS, 1% FBS, 2 mM L-glutamine (Invitrogen), 1 × 10^−4^ M nonessential amino acids (Invitrogen), 1 mM Na pyruvate (Invitrogen), 50 units of penicillin and 50 mg ml^−1^ of streptomycin) supplemented with 50 μg ml^−1^ of ascorbic acid, 10 ng ml^−1^ of BMP2, 10 ng ml^−1^ of TGF-β1 and 10 ng ml^−1^ of GDF5, which was intended to commit the cells to the chondrocytic lineage. A total of 10 ng ml^−1^ of bFGF (WAKO) was added to the chondrogenic medium from day 3 to day 14 to increase the cell proliferation. Chondrogenic cells form multilayered nodules by day 14. The nodules were physically separated from the bottom of the dishes to form particles, which were then transferred to a suspension culture in 3.5-cm petri dishes on day 14. The cells in the particles produce cartilaginous extracellular matrix, resulting in the particles becoming cartilaginous tissue in suspension culture. The culture medium was changed every 2–7 days. The hiPSC-derived cartilaginous particles on day 56 were cultured in the hypertrophic medium in the presence or absence of 300 μM pterosin B for 4 weeks.

### Real-time RT–PCR

The samples were frozen in liquid nitrogen and crushed by a Multibead shocker (Yasui Kikai). RNA was extracted by using ISOGEN (Nippon gene). For the real-time RT–PCR analyses, 500 ng of total RNA was reverse-transcribed into first-strand cDNA by using ReverTra Ace (Toyobo) and random primers. PCR amplification was performed in a reaction volume of 10 μl containing 1 μl cDNA and 5 μl SYBR FAST qPCR Master Mix (Kapa Biosystems) using 7900HT (Life Technologies). The primers used are listed in Supplementary Tables 4 and 5.

### Sequence analysis of mRNA-seq

Primary chondrocytes of *Col11a2-CreER; Sik3*^*flox/flox*^ mice were cultured in DMEM, 10% FBS medium for 5 days on a culture dish. Then, the chondrocytes were centrifuged and incubated in tubes for 3 days. The resulting cell pellets were incubated in the same medium in the presence or absence of 300 μM pterosin B for another 4 weeks. RNAs were extracted from the cultured chondrocyte pellets using QIAzol Lysis Reagent (Qiagen) and miRNeasy Mini Kit (Qiagen). RNAs were also extracted from various mouse tissues.

A 100 ng of total RNA was subjected to library preparation using KAPA stranded mRNA-seq Kits (KAPA Biosystems, Woburn, MA), according to the manufacturer's instruction. The libraries were sequenced in 100-cycle single-read mode of HiSeq2500. All sequence reads were extracted in FASTQ format using BCL2FASTQ Conversion Software 1.8.4 in the CASAVA 1.8.2 pipeline. The sequence reads were mapped to mm9 reference genes, using Tophat v2.0.14. Gene expression values were calculated and normalized using rpkmforgenes (10 December 2012). Averages of gene expression values from triplicate cultured chondrocyte pellet samples were calculated. Two-way hierarchical clustering of gene expressions in cultured chondrocyte pellets (*n*=3) and tissues (*n*=1) was conducted using the Euclidian distance and the ward method of hclust function in R3.2.1.

### Gene Ontology and pathway analysis

Genes differentially expressed between control and pterosin B-treated cells were statistically selected using DEseq2 software (|log2FC|>1, FDR<0.05). GO terms over-represented in the gene list selected by DEseq2 were retrieved by hypergeometric tests using GOstats 2.36.0 of R3.2.2 with the annotation packages GO.db 3.2.2 and org.Mm.eg.db 3.2.3. Pathway analysis was performed by hypergeometric tests of differentially expressed genes using KEGGprofile 1.12.0 of R3.2.2 with the annotation packages KEGG.db 3.2.2 and org.Mm.eg.db 3.2.3.

### Cell culture

HEK293FT cells (Invitrogen) were grown in DMEM containing 10% FBS. ATDC5 cells (Riken Cell Bank) were cultured with DMEM/Nutrient F-12 Ham (Sigma) containing 5% FBS. GAL4-MEF2C and GAL4-CRTC2 luciferase reporter assays were performed as described previously^39^.

### Microscope

Images were acquired on an inverted microscope (Eclipse Ti; Nikon) equipped with cameras (DS-Fi1; Nikon and C4742-80-12AG; Hamamatsu photonics), the NIS Elements software program (Nikon) and BZ-9000 (Keyence).

### Immunoblot analysis

Primary chondrocytes were lysed in RIPA buffer containing protease inhibitor and phosphatase inhibitor (both Roche) and subjected to SDS–polyacrylamide gel electrophoresis. The separated proteins were then electroblotted and immunostained with rabbit anti-Sik3 antibody (1:500, Abcam), rabbit anti-phosphorylated HDAC4 (Ser246)/HDAC5 (Ser259)/HDAC7 (Ser155) antibody (Cell Signaling, 1:600), rabbit anti-HDAC4 antibody (Abcam, 1:250), rabbit anti-CRTC1 (Novus Biologicals, 1:1,000) and rabbit anti-GAPDH (Santa Cruz, 1:400). The ECL system and LAS3000 (both GE Healthcare) were used for chemiluminescent immunodetection.

Full blotting images corresponding to the immunoblottings shown in the main and Supplementary Figures are provided as Supplementary Fig. 9.

### Statistical analysis

Data are shown as averages and standard deviations. We used analysis of variance followed by the Tukey–Kramer *post hoc* test. In some experiments, the Student's *t*-test (two-sided) was used when homogenous variances were assumed (*P*>0.05) by the F test, and Welch's *t*-test was used when homogenous variances were not assumed (*P*<0.05) by the F test. In osteoarthritis model experiments, score values were analysed using nonparametric statistical methods (Mann–Whitney test). *P* values <0.05 were considered statistically significant.

## Additional information

**Accession codes:** mRNA-Seq data have been deposited in the Gene Expression Omnibus under accession code GSE75995.

**How to cite this article:** Yahara, Y. *et al*. Pterosin B prevents chondrocyte hypertrophy and osteoarthritis in mice by inhibiting Sik3. *Nat. Commun.* 7:10959 doi: 10.1038/ncomms10959 (2016).

## Supplementary Material

Supplementary Information Supplementary Figures 1-9 and Supplementary Tables 1-5.
