# Corrigendum: Pterosin B prevents chondrocyte hypertrophy and osteoarthritis in mice by inhibiting Sik3

**DOI:** 10.1038/ncomms12117

**Published:** 2016-06-27

**Authors:** Yasuhito Yahara, Hiroshi Takemori, Minoru Okada, Azuma Kosai, Akihiro Yamashita, Tomohito Kobayashi, Kaori Fujita, Yumi Itoh, Masahiro Nakamura, Hiroyuki Fuchino, Nobuo Kawahara, Naoshi Fukui, Akira Watanabe, Tomoatsu Kimura, Noriyuki Tsumaki

*Nature Communications* 7: Article number: 10959 10.1038/ncomms10959 (2016); Published: 03242016; Updated: 06272016

In this Article, there is an error in the labelling of the western blot in Fig. 3f. The labels indicating the presence of pterosin B in each lane should read ‘−++ +'. The correct version of Fig. 3 appears below.


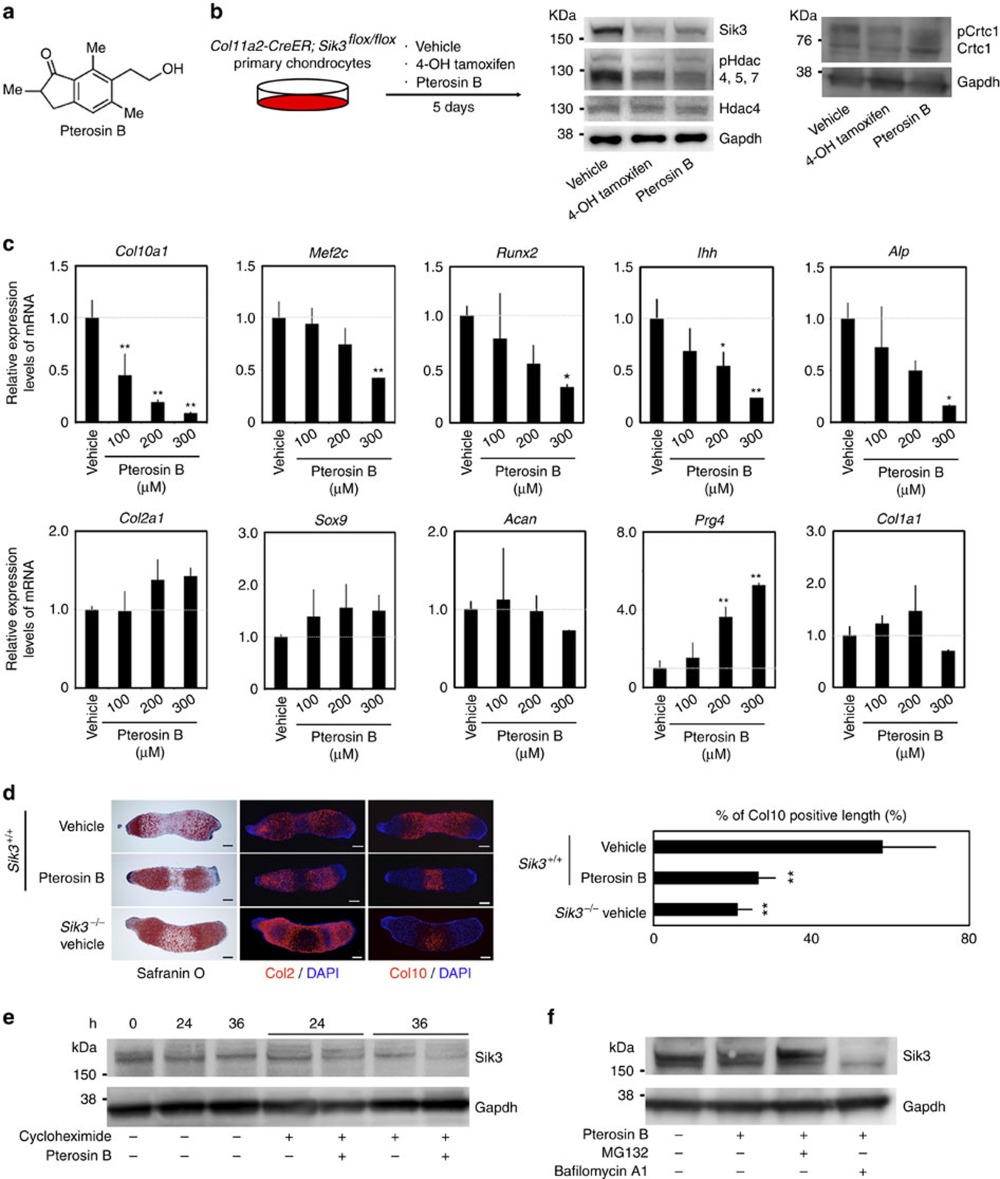


**Figure 3**

