# Supplementary material for: Pterosin B prevents chondrocyte hypertrophy and osteoarthritis in mice by inhibiting Sik3
Source: Nat Commun. 2016 Mar 24;7:10959. doi: 10.1038/ncomms10959 (PMC4820810; doi:10.1038/ncomms10959)
Supplement: Supplementary Information — Supplementary Figures 1-9 and Supplementary Tables 1-5. [file ncomms10959-s1.pdf]

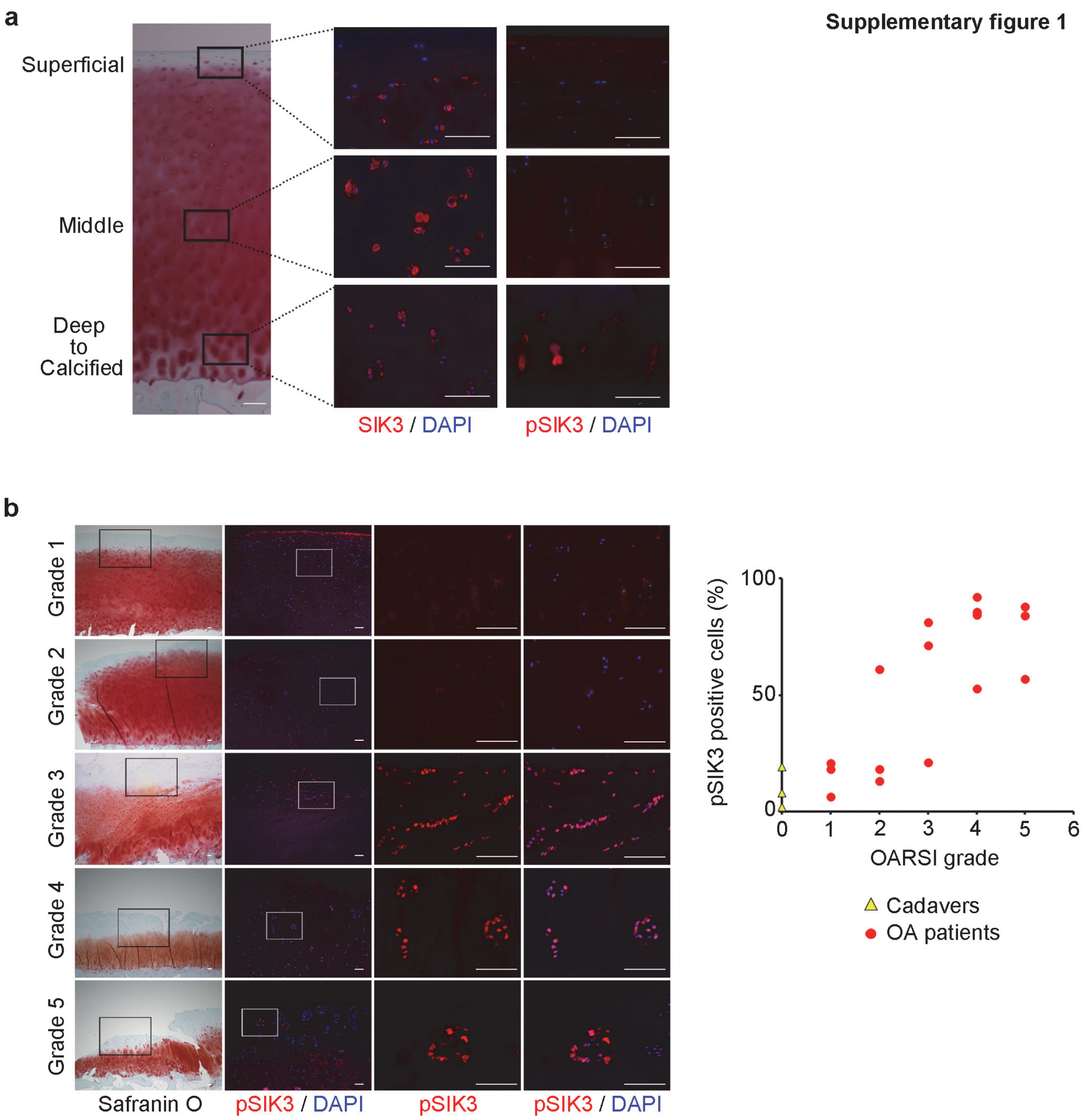

Supplementary Figure 1. Expression of phospho-SIK3 in normal and osteoarthritic articular cartilage in the knee.

(a) Semiserial histological sections of normal cartilage were stained with safranin O-fast green-iron hematoxylin and immunostained using anti-SIK3 and anti-pSIK3 antibodies. Scale bars, 100 μm

(b) We assessed safranin O-stained sections with the OARSI cartilage osteoarthritis

histopathology grading system. A representative image of sections with each OARSI grade is shown. Boxed regions are shown in the respective right panels. Scale bars, 100 μm. The graph shows the percentage of cells expressing pSIK3 per total cell number in the superficial layer.

Supplementary figure 2

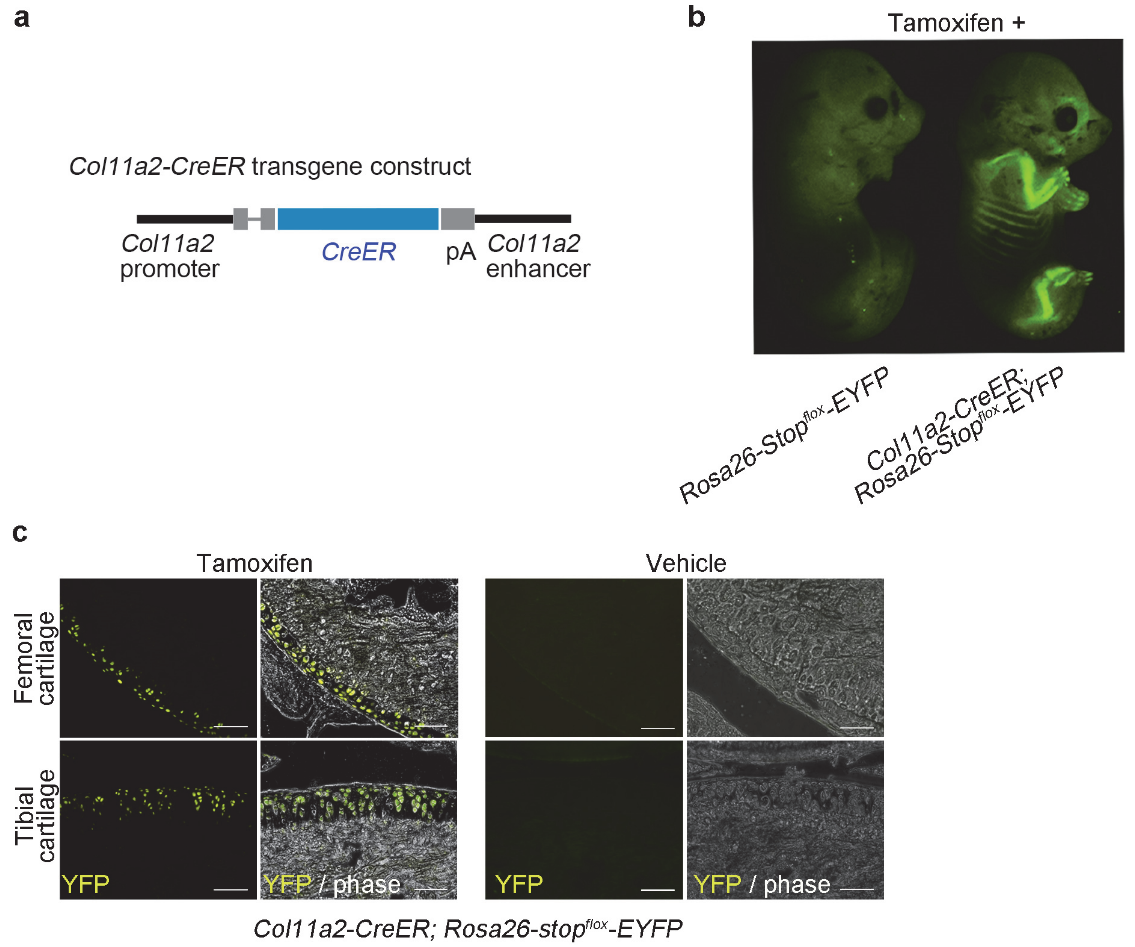

Supplementary Figure 2. Generation of *Col11a2-CreER* transgenic mice.

(a) Schematic representation of the *Col11a2-CreER* transgene construct. The *CreER* sequence was linked to *Col11a2* promoter/enhancer sequences. *pA*, polyadenylation signal sequence.

(b) A pregnant *Col11a2-CreER* mouse that had been mated with a *Rosa26-stop^{flox}-EYFP* male mouse was injected with tamoxifen at 12.5 dpc and 13.5 dpc, and sacrificed at 14.5 dpc. Embryos were observed under a fluorescence microscope using a GFP filter.

(c) 12-week old *Col11a2-CreER*; *Rosa26-stop^{flox}-EYFP* mice were injected with tamoxifen or vehicle daily for 5 consecutive days and sacrificed at 16-weeks old. Knee joints were harvested and subjected to immunohisctochemical analysis for YFP expression. Scale bars, 50 μm. The images are representative of two independent experiments.

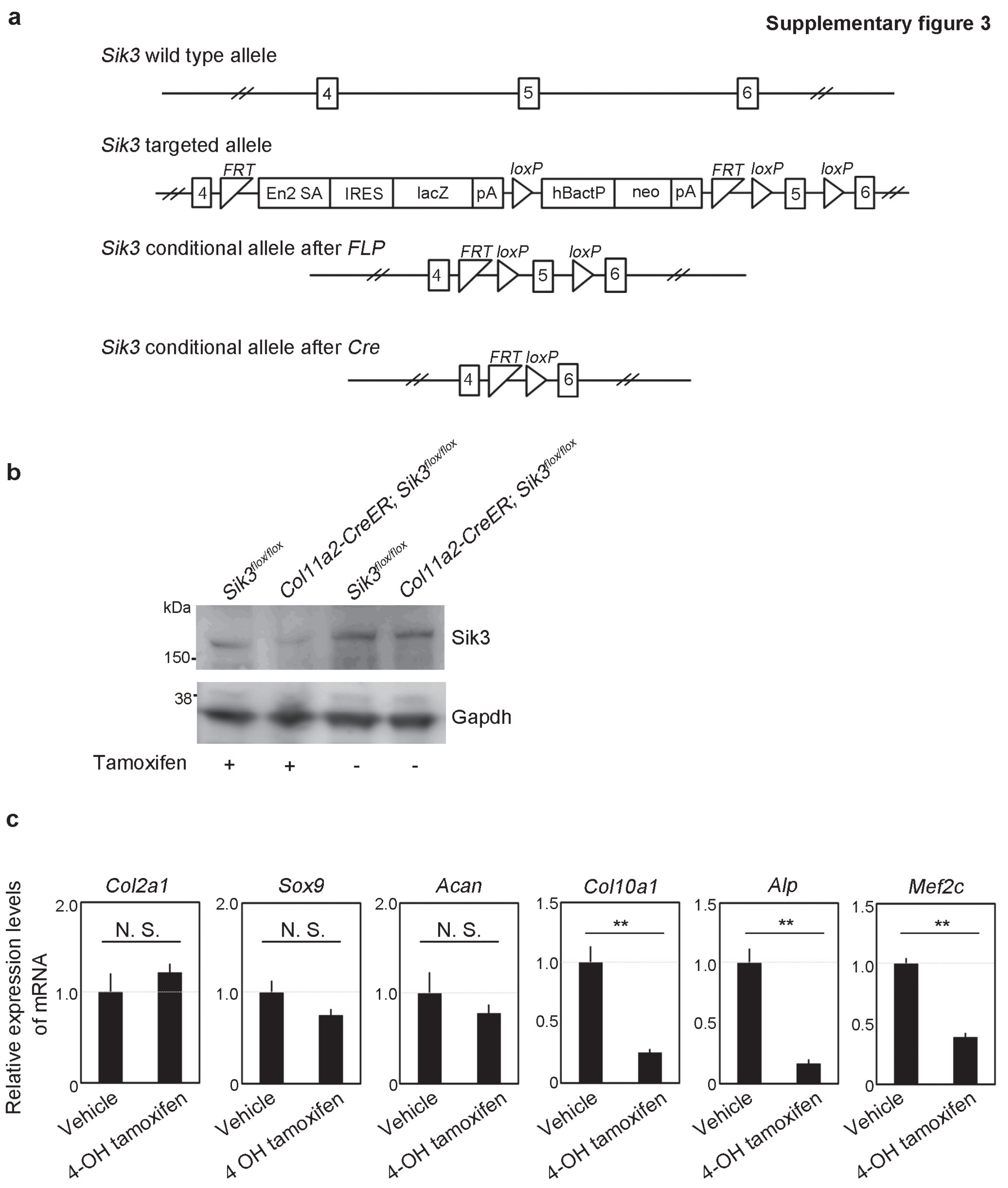

Supplementary Figure 3. Generation of tamoxifen-inducible *Sik3* conditional knockout mice.

(a) Gene targeting strategy. Structure of the *Sik3*$^{tm1a\ (EUCOMM)\ Hmgu}$ allele (modified from the

EUCOMM manual).

(b) Tamoxifen-induced Sik3 knockout in chondrocytes in *Col11a2-CreER; Sik3*$^{flox/flox}$ mice. After treatment with or without tamoxifen, as indicated at the bottom of the membrane, mice with the genotypes indicated above were sacrificed at 7-days old. Epiphyseal cartilage of the knees and femoral heads was harvested and subjected to western blot analysis.

(c) Primary chondrocytes from *Col11a2-CreER; Sik3*$^{flox/flox}$ mice were cultured in the presence or absence of 4-OH tamoxifen and subjected to pellet cultured for 4 weeks. Real-time RT-PCR expression analysis. The error bars denote means ± s.d. n = 3 pellets. **P < 0.01 and N. S., not significantly different by the *t*-test.

Supplementary figure 4

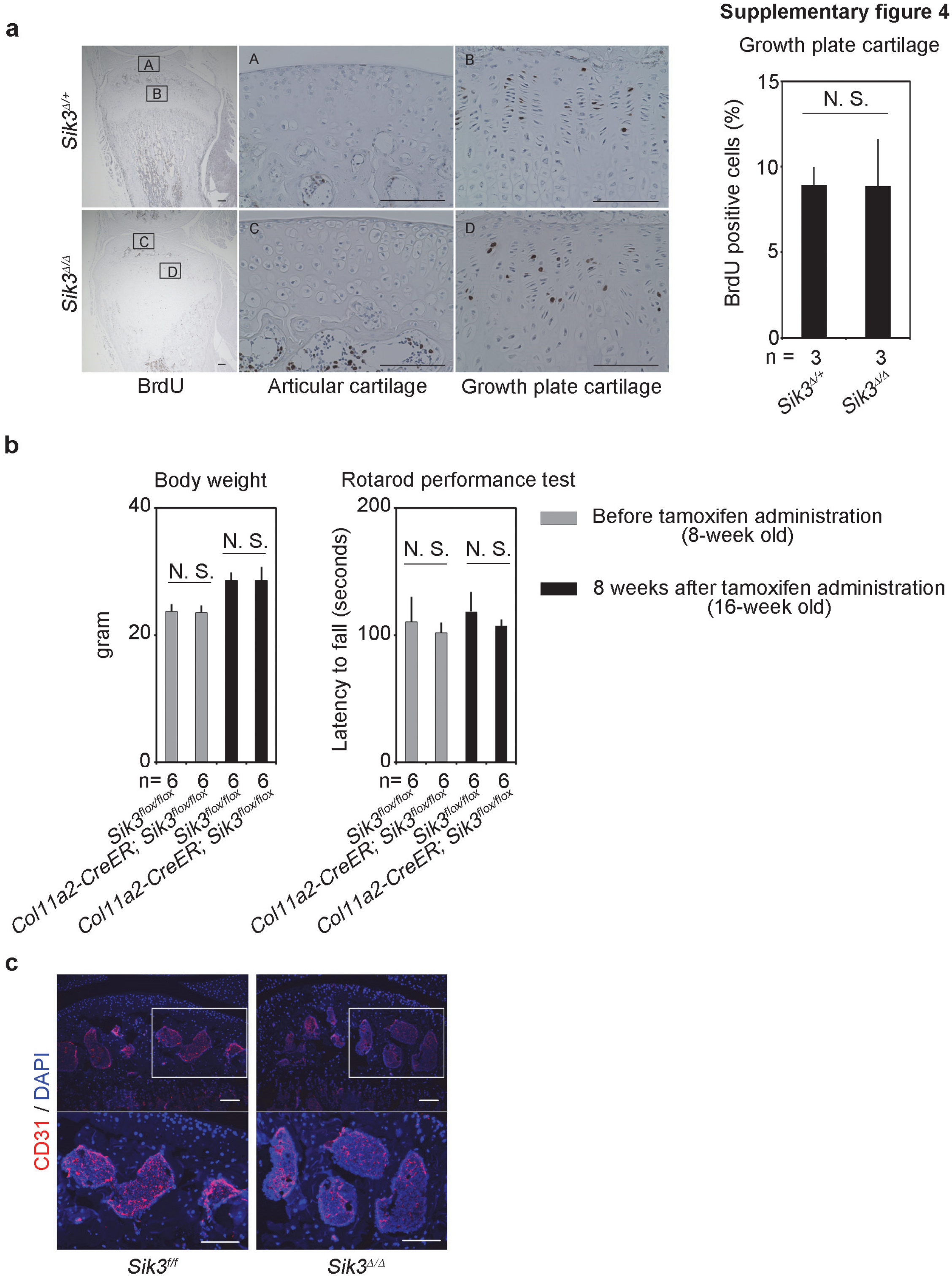

Supplementary Figure 4. BrdU labelling of proximal tibia, body weight and rotarod performance, and analysis of CD31 expression.

(a) We injected *Col11a2-CreER; Sik3$^{flox/flox}$*; *Rosa26-stop^{flox}-EYFP* mice with tamoxifen at 2-weeks old and sacrificed them at 4-weeks old. BrdU were injected 3 hours before sacrifice. Numbers of mice examined are indicated at the bottom of the graphs.

(b) 8-week old *Sik3$^{flox/flox}$* and *Col11a2-CreER; Sik3$^{flox/flox}$* mice were subjected to body weight measurements and the rotarod performance test. The mice were injected with tamoxifen and were subjected to body weight measurements and the rotarod performance test again 8 weeks later (16-weeks old). Error bars denote means ± s.d. Numbers of mice examined are indicated at the bottom of the graphs.

N. S., not significantly different by the *t*-test.

(c) 7-week old *Sik3$^{flox/flox}$* and *Col11a2-CreER; Sik3$^{flox/flox}$* mice were treated with tamoxifen for 5 days, subjected to sham operation, and sacrificed at 16-weeks old. Knee joints were harvested and subjected to immunohistochemical analysis using anti-CD31 antibody (red). Blue color is DAPI. Images of subchondral bone areas in the proximal tibia are shown. Scale bars, 100 μm. The images are representative of two independent experiments.

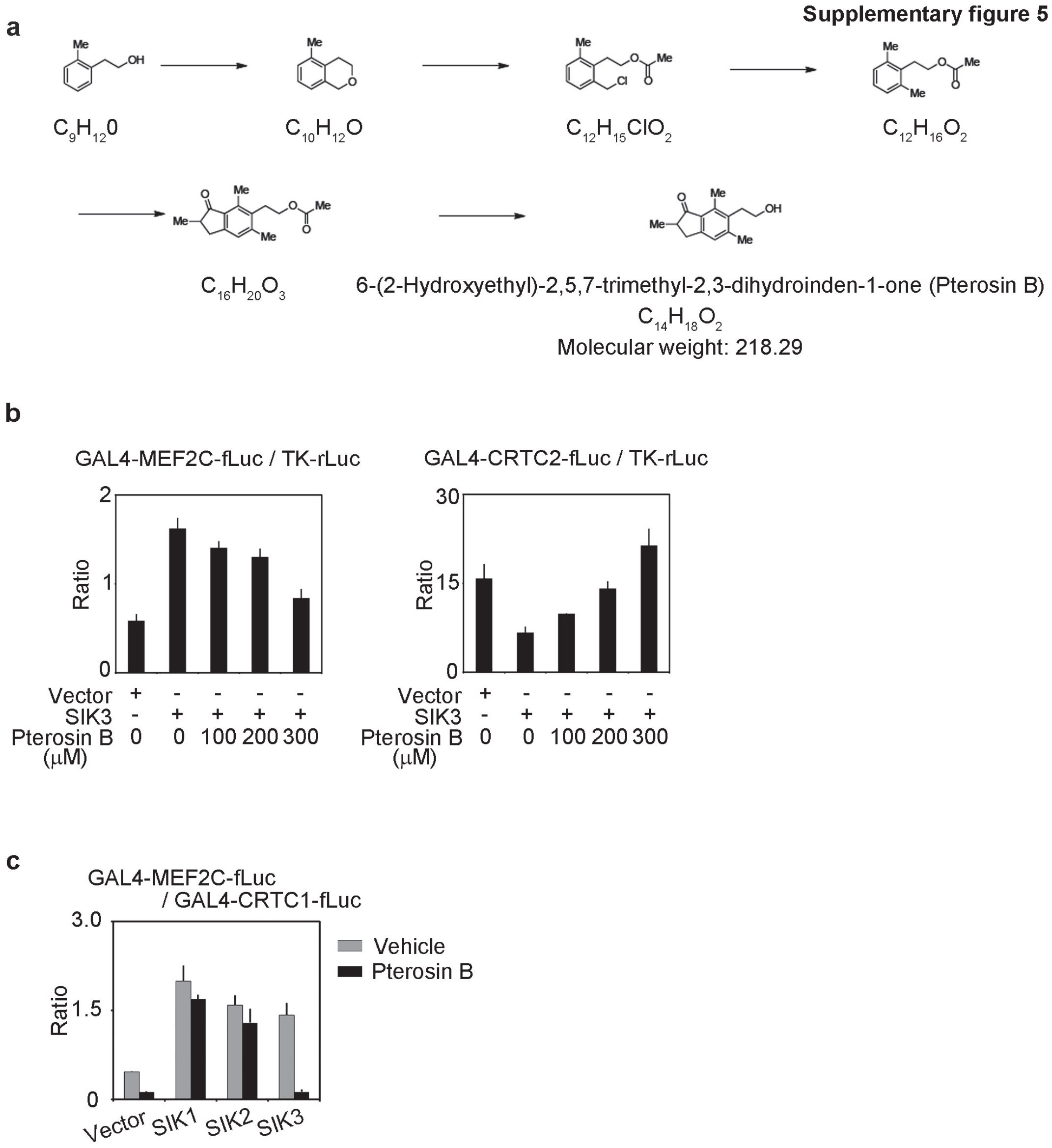

Supplementary Figure 5. Biological activity of synthesized pterosin B.

(a) Method for the synthesis of pterosin B. Reaction steps are shown.

(b) MEF2 or CRTC2 activities were measured using the GAL4-based luciferase reporter system in HEK293 cells in the presence or absence of pterosin B. n = 2.

(c) ATDC5 cells were transformed with a MEF2 or CRTC1 reporter together with SIK1-3 expression vectors and treated with pterosin B (300 μM). The fold differences in the reporter activities by the pterosin B treatment are indicated (Means ± SD). n = 2.

Supplementary figure 6

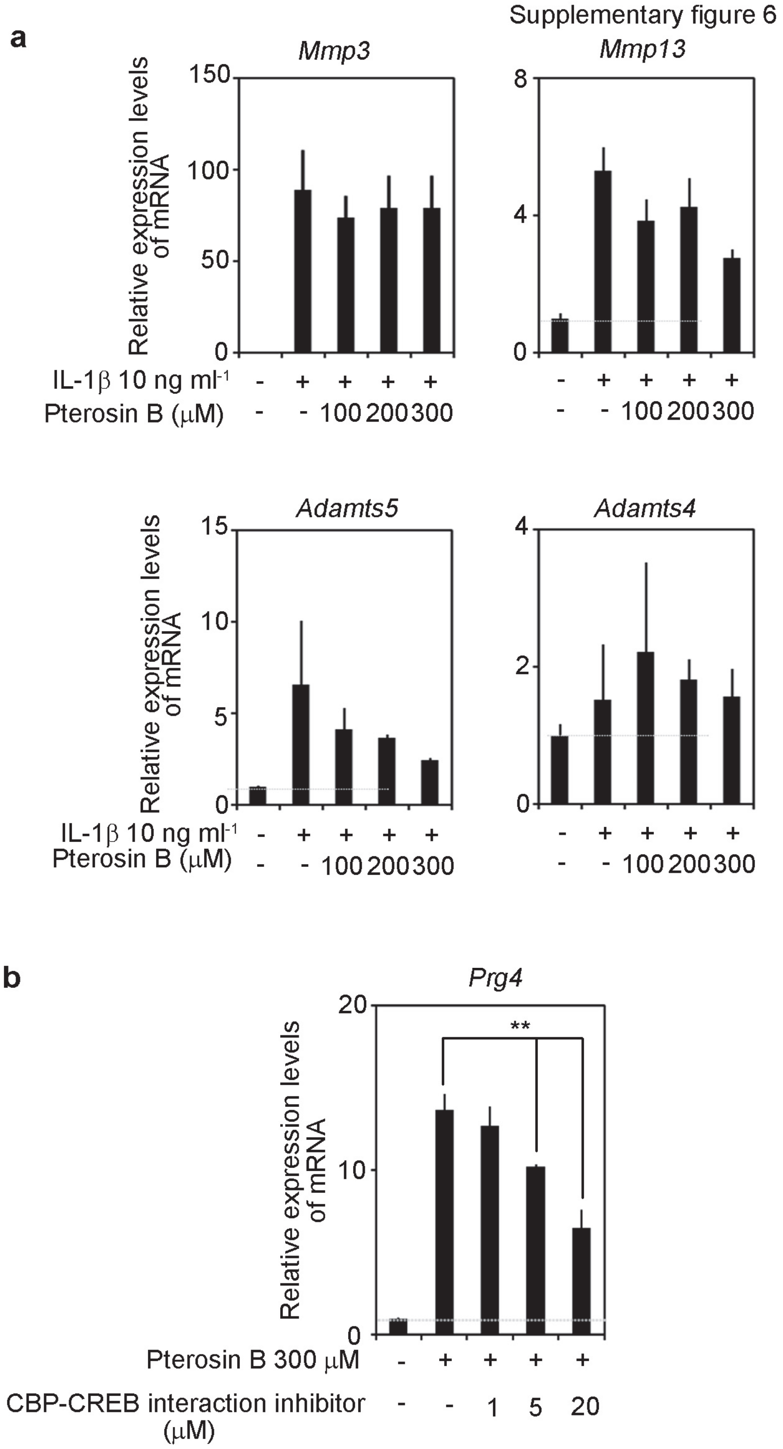

Supplementary Figure 6. Effects of pterosin B on the expression of cartilage genes.

(a) mRNA expression levels of catalytic enzymes in pellet culture of mouse primary chondrocytes. Pellet cultures were performed for 4 weeks. Subsequently, pellets were treated with 10 ng/ml interleukin 1 (IL-1β) in various concentrations of pterosin B (0, 100, 200, 300 μM) for 24 hrs. Error bars denote means ± s.d. n = 3 pellets. Not significant difference by the Tukey–Kramer post-hoc test.

(b) *Prg4* mRNA expression in mouse primary chondrocytes. Pellets of mouse primary chondrocytes were cultured in hypertrophic medium in the absence or presence of 300 μM pterosin B and various concentrations (1, 5, 20 μM) of CBP-CREB interaction inhibitor for two weeks. Error bars denote means ± s.d. n = 3 pellets. **P < 0.01 by the Tukey–Kramer post-hoc test.

Supplementary figure 7

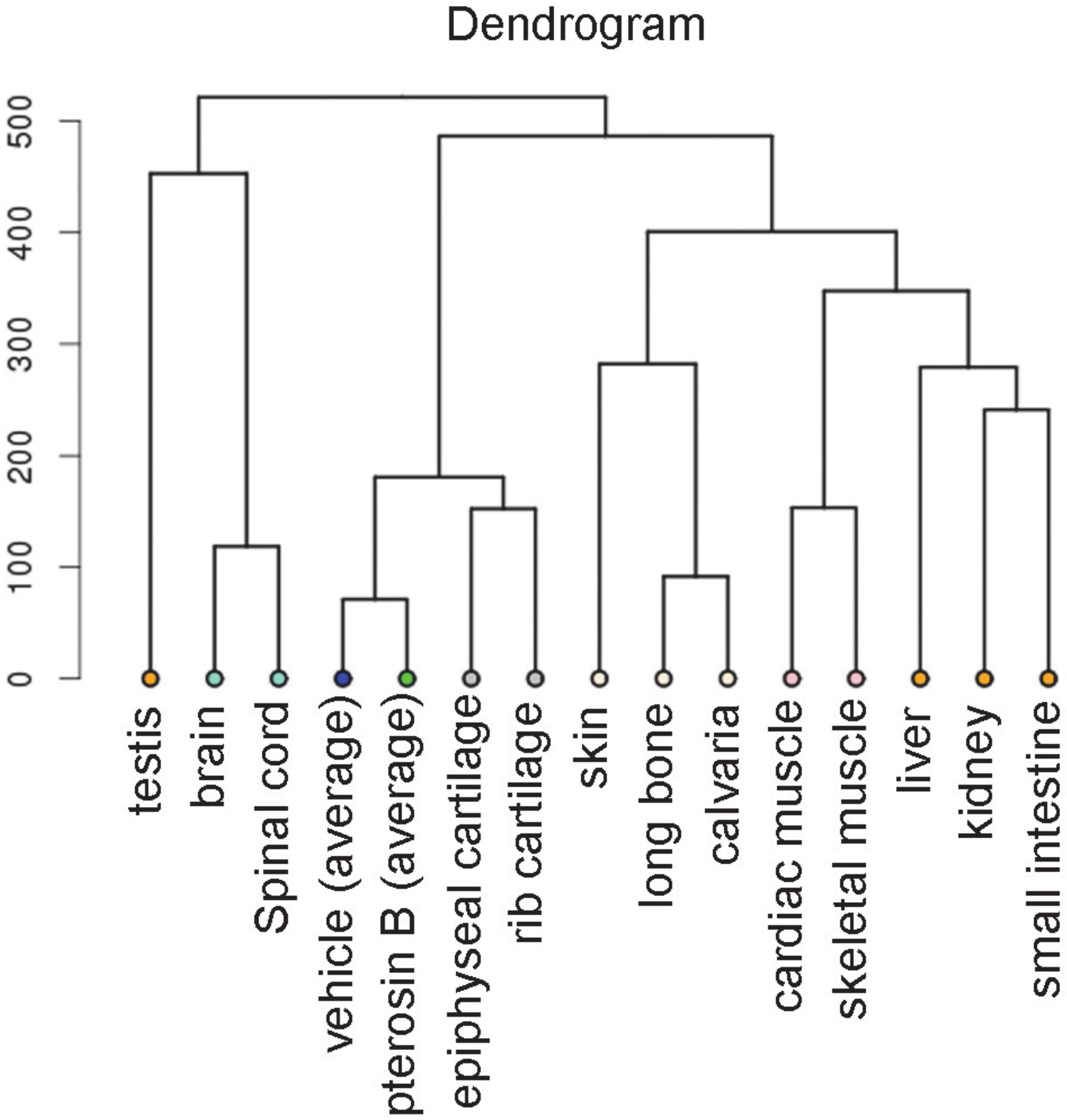

Supplementary Figure 7. RNA-sequencing analysis on mouse chondrocyte pellet culture in the absence or presence of pterosin B and various mouse tissues. Cluster analysis based on whole mRNA transcripts is shown. *Vehicle*, mouse chondrocyte pellets cultured in the absence of pterosin B. *Pterosin B*, mouse chondrocyte pellets cultured in the presence of pterosin B.

Supplementary figure 8

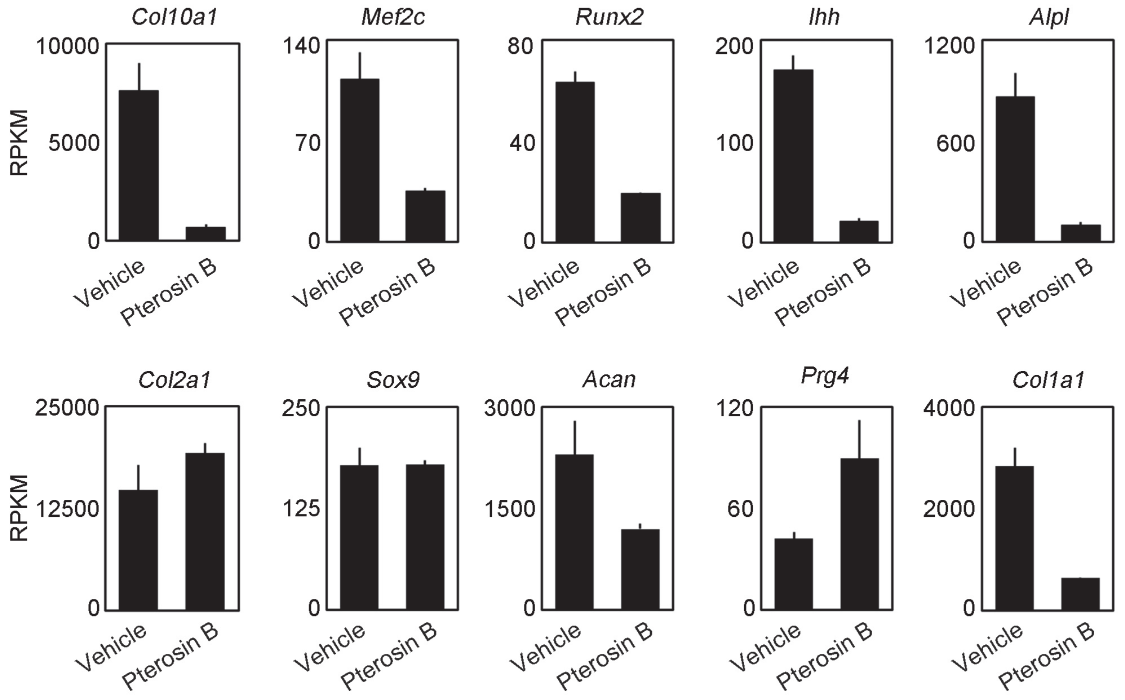

Supplementary Figure 8. Expression of marker genes in mouse chondrocyte pellet culture in the absence or presence of 300 μM pterosin B based on RNA-sequencing analysis. *RPKM*, reads per kirobase of exon per million sequence reads. *Vehicle*, mouse chondrocyte pellets cultured in the absence of pterosin B. *Pterosin B*, mouse chondrocyte pellets cultured in the presence of pterosin B. Error bars denote means ± s.d. n = 3 pellets.

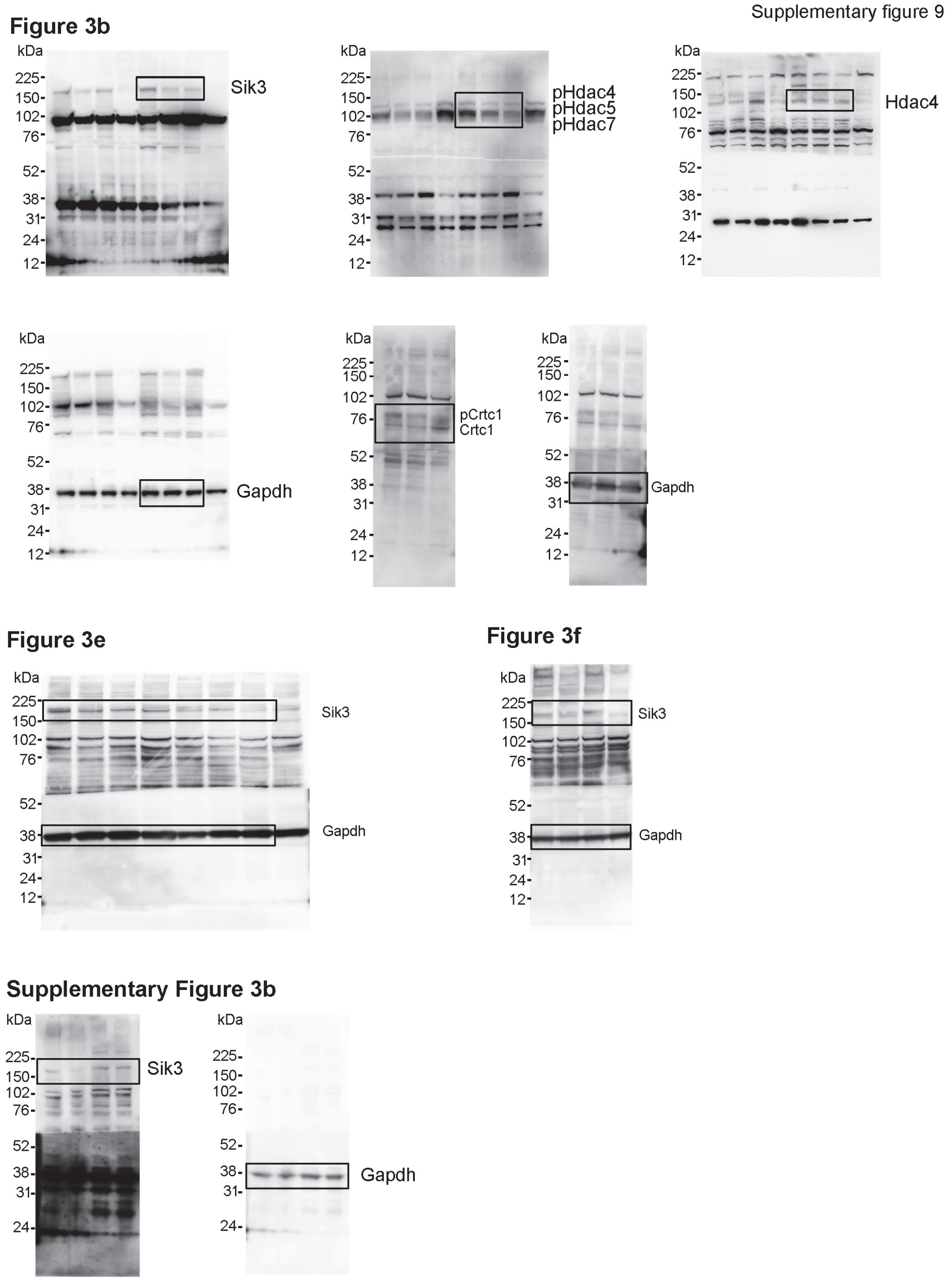

Supplementary Figure 9. Full scans of western blots related to respective figures as indicated. The protein of interest being detected is labeled to the right of each blot.

Supplementary Table 1. Gene Ontology analysis of genes down-regulated by pterosin B treatment

GOBPID	Term	*P* value	Count	Size
GO:0030282	bone mineralization	5.17E-11	19	96
GO:0031214	biomineral tissue development	9.46E-11	21	123
GO:0030500	regulation of bone mineralization	8.58E-09	14	67
GO:0097529	myeloid leukocyte migration	1.08E-08	19	130
GO:0030595	leukocyte chemotaxis	1.56E-08	20	147
GO:0070167	regulation of biomineral tissue development	2.28E-08	14	72

Supplementary Table 2. Articular cartilage from OA patients

No.	Age	Sex	OARSI grade
1	71	F	4
2	75	F	1
3	72	M	4
4	75	F	3
5	77	F	2
6	64	M	1
7	66	F	2
8	64	F	3
9	72	F	5
10	71	F	2
11	74	M	4
12	84	F	4
13	67	F	1
14	85	F	3
15	76	F	5
16	72	F	5

Supplementary Table 3. Articular cartilage from nonsymptomatic cadavers

No.	Age	Sex	OARSI grade
1	95	M	0
2	63	M	0
3	73	F	0

Supplementary Table 4. The sequence of primers for mouse genes

Primer	Sequence
Col10a1 F	TTCTGCTGCTAATGTTCTTGACC
Col10a1 R	GGGATGAAGTATTGTGTCTTGGG
Mef2c F	ACGAGGATAATGGATGAGCGT
Mef2c R	ATCAGTGCAATCTCACAGTCG
Alp F	CCAACTCTTTTGTGCCAGAGA
Alp R	GGCTACATTGGTGTTGAGCTTTT
Col2a1 F	TTGAGACAGCACGACGTGGAG
Col2a1 R	AGCCAGGTTGCCATCGCCATA
Sox9 F	TGAAGAAGGAGAGCGAGGAGGA
Sox9 R	ATCTCCCCCAACGCCATCTT
Prg4 F	TGGAGTGCTGTCCTGATTTCAAGAG
Prg4 R	GGTGATTTGGGTGAGCGTTTGGTA
Runx2 F	GACTGTGGTTACCGTCATGGC
Runx2 R	ACTTGGTTTTTCATAACAGCGGA
Ihh F	CTCTTGCCTACAAGCAGTTCA
Ihh R	CCGTGTTCTCCTCGTCCTT
Acan F	CCCTCGGGCAGAAGAAAGAT
Acan R	CGCTTCTGTAGCCTGTGCTTG
*Col1a1*F	GCAACAGTCGCTTCACCTAC
*Col1a1*R	GTGGGAGGGAACCAGATTG
Mmp3 F	GGCCTGGAACAGTCTTGGC
Mmp3 R	TGTCCATCGTTCATCATCGTCA
Mmp13 F	TGTTTGCAGAGCACTACTTGAA
Mmp13 R	CAGTCACCTCTAAGCCAAAGAAA
Adamts5 F	CCCAGGATAAAACCAGGCAG
Adamts5 R	CGGCCAAGGGTTGTAAATGG
Adamts4 F	ATGGCCTCAATCCATCCCAG
Adamts4 R	GCAAGCAGGGTTGGAATCTTTG
Gapdh F	AAGCCCATCACCATCTTCCAGGAG
Gapdh R	ATGAGCCCTTCCACAATGCCAAAG

Supplementary Table 5. The sequence of primers for human genes

Primer	Sequence
COL10A1 F	ATGCTGCCACAAATACCCTTT
COL10A1 R	GGAATGAAGAACTGTGTCTTGGT
ALP F	ACCACCACGAGAGTGAACCA
ALP R	CGTTGTCTGAGTACCAGTCCC
COL2A1 F	GTGGAGCAGCAAGAGCAA
COL2A1 R	TGTTGGGAGCCAGATTGT
SOX9 F	AGCGAACGCACATCAAGAC
SOX9 R	CTGTAGGCGATCTGTTGGGG
PRG4 F	AAAGTCAGCACATCTCCCAAG
PRG4 R	GTGTCTCTTTAGCGGAAGTAGTC
GAPDH F	AATGGACAACTGGTCGTGGAC
GAPDH R	CCCTCCAGGGGATCTGTTTG
